# Supplementary material for: Early events marking lung fibroblast transition to profibrotic state in idiopathic pulmonary fibrosis
Source: Respir Res. 2023 Apr 21;24:116. doi: 10.1186/s12931-023-02419-0 (PMC10122312; doi:10.1186/s12931-023-02419-0)
Supplement: Supplementary file 1 — Additional file 1. Supplemental Data. [file 12931_2023_2419_MOESM1_ESM.pdf]

Supplemental Data

Jia, et al, "*Early events marking lung fibroblast transition to profibrotic state in idiopathic pulmonary fibrosis*"

TABLES

Supplementary Table S1. Human Samples

Experimental ID/ Sample ID	Age	Gender	Application
SC56-NOR/ 2017-025-NOR	57	F	scRNA-seq
SC59-NOR/ 2017-029-NOR	18	M	scRNA-seq
SC155-NOR-low/ 2017-105-NOR	23	F	scRNA-seq
SC156-NOR-up/ 2017-105-NOR			scRNA-seq
SC87-IPF-low/ 2017-064-IPF	70	F	scRNA-seq
SC88-IPF-up/ 2017-064-IPF			scRNA-seq
SC93-IPF-low/ 2017-067-IPF	69	M	scRNA-seq
SC94-IPF-up/ 2017-067-IPF			scRNA-seq
SC153-IPF-low/ 2017-100-IPF	69	F	scRNA-seq
SC154-IPF-up/ 2017-100-IPF			scRNA-seq
Figure 4D_control/ Core-025-NOR	57	F	Immunofluorescence
Figure 4D_IPF donor-2 /Core-022-IPF-low	61	F	Immunofluorescence
Figure 4D_IPF donor-1/ Core-067-IPF-low	69	M	Immunofluorescence
Core-015-NOR	23	F	Cell culture, Immunofluorescence
Core-009-NOR	68	F	Cell culture, Immunofluorescence
Core-31-IPF-low	64	F	Cell culture, Immunofluorescence
Core-14-IPF-up	66	M	Cell culture, Immunofluorescence

Supplementary Table S2. Immunostaining antibody list

Primary antibody	Secondary antibody
MOXD1 (1:100) (Sigma)<br>MOXD1 (1:100) Bioss	Anti rabbit-647 (1:500) (Thermo Fisher)<br>Anti-rabbit Alexa Fluor 647 goat (1:300)
α-SMA (1:1000) (DAKO)<br>α-SMA (1:200) (Santa Cruz Biotechn)	Anti mouse-555 (1:500) (ThermoFisher)<br>Anti-mouse Alexa Fluor 555 goat (1:300)
P-p53 (1:400) (Cell Signaling)	Anti mouse-555 (1:500) (ThermoFisher)
P16 (1:100) (BD)	Anti mouse-555 (1:500) (ThermoFisher)
PDGFRα (1:100) eBioscience	Anti-rat Alexa Fluor 488 goat (1:300)

Supplementary Table S3. Differentially expressed genes (upregulated) in each of the four fibroblasts subclusters. The top 16 genes (according to average fold change) are listed in bold.

**GeneSymbol**	**avg_FC**	**p_val**	**p_val_adj**	**CLUSTER**
**PTX3**	2.36587758	2.21E-122	7.43E-118	1
**CXCL8**	1.93111571	2.87E-150	9.68E-146	1
**IL6**	1.85552638	2.69E-144	9.05E-140	1
**CXCL2**	1.76951581	9.32E-146	3.14E-141	1
**CCL2**	1.76467289	6.38E-92	2.15E-87	1
**CXCL1**	1.52891309	1.35E-103	4.55E-99	1
**SOD2**	1.3721351	7.04E-137	2.37E-132	1
**ICAM1**	1.36542222	8.41E-159	2.84E-154	1
**GADD45B**	1.28125224	8.62E-97	2.90E-92	1
**THBS1**	1.25051032	5.84E-132	1.97E-127	1
**CXCL3**	1.23154164	1.52E-140	5.11E-136	1
**IER3**	1.21331222	3.37E-98	1.13E-93	1
**NFKBIA**	1.17631579	9.16E-120	3.09E-115	1
**ADM**	1.08866149	1.01E-100	3.41E-96	1
**MT1A**	1.04788844	3.88E-86	1.31E-81	1
**CH25H**	1.01896576	1.68E-81	5.66E-77	1
NAMPT	0.99202619	9.70E-165	3.27E-160	1
GPX3	0.98838742	5.22E-88	1.76E-83	1
SOCS3	0.92881241	1.03E-82	3.47E-78	1
RGCC	0.89812617	8.16E-56	2.75E-51	1
HSPA1B	0.89704349	1.25E-34	4.23E-30	1
G0S2	0.87825078	4.12E-29	1.39E-24	1
CYR61	0.87518304	2.58E-33	8.68E-29	1
SRGN	0.86303231	1.03E-134	3.46E-130	1
C11orf96	0.83416118	2.98E-68	1.00E-63	1
SAT1	0.83244217	2.60E-104	8.76E-100	1
IRF1	0.83124369	1.05E-104	3.53E-100	1
MT1M	0.81106881	6.24E-62	2.10E-57	1
HES1	0.7957278	1.24E-66	4.19E-62	1
MT2A	0.77667449	3.20E-63	1.08E-58	1
CEBPB	0.76957941	1.15E-108	3.88E-104	1
LMCD1	0.75086255	1.16E-28	3.89E-24	1
HSPA1A	0.74120463	4.46E-20	1.50E-15	1
RGS2	0.74043228	4.75E-69	1.60E-64	1
SFTPC	0.7344558	9.98E-120	3.36E-115	1

BIRC3	0.7294614	3.19E-149	1.07E-144	1
RGS16	0.71386118	1.27E-81	4.29E-77	1
MYC	0.7077958	2.24E-60	7.53E-56	1
MALAT1	0.69209451	6.02E-73	2.03E-68	1
CSRP2	0.68847529	3.72E-95	1.25E-90	1
AREG	0.67241131	1.35E-113	4.55E-109	1
ADAMTS4	0.671993	2.73E-118	9.20E-114	1
CDKN1A	0.67109262	4.87E-111	1.64E-106	1
TNFAIP3	0.66392633	5.92E-130	1.99E-125	1
CYP4B1	0.66198684	3.44E-80	1.16E-75	1
NFKBIZ	0.66044285	1.84E-103	6.21E-99	1
TNFAIP2	0.65820221	9.14E-108	3.08E-103	1
CCNL1	0.65382153	7.32E-139	2.47E-134	1
PLAUR	0.64075834	6.10E-129	2.06E-124	1
PTGS2	0.64017027	9.74E-82	3.28E-77	1
CEBPD	0.61413236	9.03E-53	3.04E-48	1
JUNB	0.60976395	3.58E-55	1.21E-50	1
PNRC1	0.6042063	1.18E-99	3.97E-95	1
NABP1	0.60278856	1.80E-159	6.05E-155	1
MACF1	0.57549836	9.54E-71	3.22E-66	1
MAFF	0.56942318	1.63E-130	5.48E-126	1
ZFP36	0.56647645	7.20E-44	2.43E-39	1
HLA-DRA	0.55984227	1.42E-105	4.80E-101	1
FABP4	0.5468096	2.36E-114	7.94E-110	1
FIGF	0.54660362	3.03E-59	1.02E-54	1
FMO2	0.54558637	4.79E-53	1.61E-48	1
CLDN5	0.54533667	2.34E-127	7.89E-123	1
PPP1R15A	0.54394795	1.58E-91	5.34E-87	1
SAA1	0.54240319	1.67E-103	5.64E-99	1
REL	0.5300019	1.40E-157	4.73E-153	1
CRISPLD2	0.5271223	4.71E-40	1.59E-35	1
S100A9	0.52191081	3.38E-136	1.14E-131	1
CCL4	0.50811931	1.20E-98	4.03E-94	1
XBP1	0.50455752	5.18E-114	1.75E-109	1
MT1X	0.50156475	5.47E-10	1.84E-05	1
TNFAIP6	0.50085669	4.37E-13	1.47E-08	1
PRELP	0.49882153	1.64E-13	5.54E-09	1
THUMPD3-AS1	0.49736298	6.25E-129	2.11E-124	1
ARID5B	0.49239879	2.27E-36	7.66E-32	1

HLA-E	0.49109298	1.08E-116	3.64E-112	1
C8orf4	0.48986074	2.91E-24	9.81E-20	1
USP53	0.48968097	1.11E-66	3.76E-62	1
ARID5A	0.48384306	1.20E-191	4.03E-187	1
IER2	0.48086841	2.58E-30	8.69E-26	1
ZC3H12A	0.4796793	1.77E-150	5.97E-146	1
ANGPTL4	0.47474595	5.68E-56	1.92E-51	1
MCL1	0.47191571	2.64E-119	8.89E-115	1
KDM6B	0.46587959	4.90E-147	1.65E-142	1
CXCR4	0.46528447	2.40E-89	8.10E-85	1
TRIB1	0.46406927	7.13E-86	2.40E-81	1
CRYAB	0.46049081	0.00031378	1	1
BAZ1A	0.45952845	1.41E-192	4.74E-188	1
CD52	0.45758224	1.24E-100	4.18E-96	1
SCGB1A1	0.45485819	2.17E-43	7.32E-39	1
MAP3K8	0.45437703	5.08E-150	1.71E-145	1
TM4SF1	0.44717669	2.75E-71	9.28E-67	1
DDX21	0.44629363	6.30E-151	2.12E-146	1
ETS2	0.44209865	6.46E-148	2.18E-143	1
GEM	0.43882813	2.04E-15	6.88E-11	1
TNFRSF12A	0.43872326	7.69E-59	2.59E-54	1
SERTAD1	0.43111565	2.35E-80	7.93E-76	1
BTG1	0.43021595	2.93E-72	9.89E-68	1
CYP1B1	0.43011311	1.13E-13	3.81E-09	1
GPM6B	0.42694038	1.05E-48	3.53E-44	1
HSPE1	0.42662256	5.39E-75	1.81E-70	1
ADAMTS1	0.42660966	6.26E-41	2.11E-36	1
ZNF331	0.42229387	9.29E-148	3.13E-143	1
WTAP	0.42176563	5.94E-130	2.00E-125	1
DNAJA1	0.421392	9.42E-79	3.17E-74	1
S100A8	0.42021121	2.08E-111	7.02E-107	1
SFTPB	0.41954204	7.27E-113	2.45E-108	1
SOX4	0.41691108	5.33E-28	1.80E-23	1
CD14	0.41574186	2.38E-123	8.03E-119	1
CD74	0.41416248	7.58E-59	2.56E-54	1
C1QA	0.4123415	2.95E-107	9.92E-103	1
LYZ	0.41064819	3.34E-111	1.13E-106	1
MT-ND3	0.40762542	3.47E-29	1.17E-24	1
CSF3	0.40215208	3.02E-143	1.02E-138	1
FCER1G	0.40134922	3.67E-122	1.24E-117	1

CITED2	0.39977401	9.39E-43	3.16E-38	1
SPINT2	0.39849867	3.96E-79	1.33E-74	1
TCEB1	0.3924259	1.37E-72	4.61E-68	1
C1QB	0.39181774	1.55E-108	5.24E-104	1
MIR22HG	0.38677725	9.58E-140	3.23E-135	1
CREM	0.3834248	2.19E-161	7.37E-157	1
RSRP1	0.38066411	1.31E-94	4.40E-90	1
ADAMTS9	0.37619347	2.28E-111	7.68E-107	1
KLF10	0.37572671	2.08E-45	7.02E-41	1
SLC2A3	0.37408946	1.45E-76	4.89E-72	1
EPAS1	0.37396926	4.04E-103	1.36E-98	1
CFLAR	0.37307352	5.54E-121	1.87E-116	1
BAG3	0.37206327	1.84E-73	6.21E-69	1
CD69	0.3708325	4.26E-113	1.43E-108	1
CYCS	0.37061349	2.21E-117	7.44E-113	1
DDX3X	0.3678882	2.38E-148	8.02E-144	1
SCN7A	0.36390954	1.64E-07	0.0055344	1
NEDD9	0.36251133	5.06E-83	1.70E-78	1
SLC25A37	0.36199209	4.21E-124	1.42E-119	1
LDLR	0.3615974	2.44E-149	8.23E-145	1
H3F3B	0.36068897	1.59E-89	5.35E-85	1
NR4A2	0.36011317	5.99E-94	2.02E-89	1
LIF	0.35935812	1.38E-84	4.64E-80	1
PTGIR	0.35841496	9.17E-33	3.09E-28	1
CSRNP1	0.35832394	1.22E-78	4.10E-74	1
HBEGF	0.35702656	1.23E-147	4.15E-143	1
CDH13	0.35580477	1.89E-36	6.36E-32	1
XIST	0.3551116	3.74E-22	1.26E-17	1
IFRD1	0.35447549	9.18E-71	3.09E-66	1
TYROBP	0.35444006	1.08E-97	3.64E-93	1
CTGF	0.35263765	9.55E-10	3.22E-05	1
CTSC	0.35263654	9.18E-30	3.09E-25	1
BTG3	0.35175	2.52E-78	8.49E-74	1
LITAF	0.34932298	3.17E-50	1.07E-45	1
GMFG	0.3483106	6.22E-133	2.10E-128	1
CTD-3252C9.4	0.34444418	2.22E-72	7.48E-68	1
SRSF7	0.34439817	9.85E-69	3.32E-64	1
WSB1	0.3435438	4.35E-66	1.47E-61	1
DNAJB1	0.34042281	8.88E-18	2.99E-13	1

SON	0.3401524	3.39E-104	1.14E-99	1
VWF	0.339502	4.49E-109	1.51E-104	1
PIM1	0.33848937	1.13E-74	3.82E-70	1
FABP5	0.33772185	1.26E-80	4.25E-76	1
LAPTM5	0.33756679	1.01E-122	3.40E-118	1
SFPQ	0.33751547	5.40E-163	1.82E-158	1
HEXIM1	0.3364138	6.63E-108	2.23E-103	1
IDI1	0.33598871	6.68E-166	2.25E-161	1
HSPD1	0.33567292	2.93E-65	9.87E-61	1
HSP90AB1	0.33545442	8.71E-36	2.93E-31	1
IL7R	0.33036306	2.08E-151	7.00E-147	1
HSP90AA1	0.32971137	5.38E-24	1.81E-19	1
ATP1B3	0.32912629	5.66E-104	1.91E-99	1
APOC1	0.3284283	2.76E-61	9.31E-57	1
AOC3	0.32772526	2.47E-13	8.32E-09	1
LINC00152	0.32541792	2.64E-53	8.89E-49	1
HNRNPH1	0.32541519	2.32E-67	7.83E-63	1
PIM3	0.32516098	1.55E-121	5.22E-117	1
GPC3	0.32399375	3.71E-23	1.25E-18	1
ACKR1	0.32396986	8.75E-77	2.95E-72	1
DDX5	0.32185008	8.40E-63	2.83E-58	1
SAMSN1	0.32153248	1.98E-172	6.68E-168	1
PLIN2	0.32148155	8.86E-52	2.99E-47	1
TUBB4B	0.32073038	3.93E-79	1.32E-74	1
ELL2	0.32056713	1.25E-142	4.20E-138	1
UPP1	0.31903524	8.09E-170	2.73E-165	1
CHIC2	0.31902184	1.19E-112	4.02E-108	1
TSPYL2	0.31817	3.89E-105	1.31E-100	1
MT1E	0.31583262	8.54E-10	2.88E-05	1
INMT	0.31555403	3.33E-13	1.12E-08	1
IL1B	0.31410341	1.37E-165	4.62E-161	1
CCBE1	0.31370597	1.99E-51	6.71E-47	1
EIF5	0.31285266	6.32E-104	2.13E-99	1
EIF4A1	0.31267278	1.11E-98	3.72E-94	1
CA3	0.31148537	9.63E-42	3.24E-37	1
EIF4A3	0.30925761	1.11E-82	3.75E-78	1
VMP1	0.30846366	1.97E-45	6.64E-41	1
MIDN	0.30762275	3.68E-120	1.24E-115	1
HSPH1	0.3073917	2.47E-41	8.32E-37	1
CXCL10	0.30525174	1.35E-18	4.54E-14	1

SERPINB9	0.30504094	2.53E-158	8.52E-154	1
CCL20	0.30408159	2.95E-170	9.93E-166	1
RNASE1	0.3039461	9.59E-99	3.23E-94	1
STX11	0.30248317	1.20E-177	4.04E-173	1
ALOX5AP	0.3018099	1.60E-99	5.40E-95	1
UBC	0.3014861	3.35E-27	1.13E-22	1
HLA-DPA1	0.30029034	7.47E-92	2.52E-87	1
CRIM1	0.30008874	2.54E-88	8.54E-84	1
CD83	0.30005094	1.11E-142	3.73E-138	1
KMT2E	0.29958592	6.59E-105	2.22E-100	1
SVEP1	0.29844991	8.02E-38	2.70E-33	1
ATF3	0.29841542	1.54E-19	5.20E-15	1
SLC25A25	0.29633749	4.16E-127	1.40E-122	1
FSTL3	0.29421793	9.89E-35	3.33E-30	1
PLSCR1	0.29413666	1.73E-109	5.83E-105	1
UBE2D3	0.29411123	3.53E-118	1.19E-113	1
ID2	0.29350482	9.98E-17	3.36E-12	1
EMP2	0.29229129	9.60E-20	3.23E-15	1
MAP2K3	0.2915011	3.10E-132	1.04E-127	1
ZFAND5	0.29111983	1.85E-49	6.25E-45	1
ICAM4	0.29026084	4.63E-108	1.56E-103	1
SLIT2	0.29013777	1.73E-08	0.00058298	1
FGFR4	0.2893808	1.93E-40	6.50E-36	1
SRSF3	0.28918224	7.46E-94	2.52E-89	1
SGK1	0.28410965	1.05E-72	3.53E-68	1
FCN3	0.28356439	4.34E-95	1.46E-90	1
STAT3	0.28268956	4.51E-63	1.52E-58	1
PLK3	0.28268849	2.79E-84	9.40E-80	1
AASS	0.2820472	8.94E-65	3.01E-60	1
DNAJB9	0.2792101	3.85E-65	1.30E-60	1
CHI3L2	0.27904028	4.10E-77	1.38E-72	1
HLA-DRB5	0.27834271	3.34E-84	1.13E-79	1
RCAN1	0.27674957	7.49E-69	2.52E-64	1
MT1G	0.27672788	2.86E-84	9.62E-80	1
NFKB1	0.27623641	5.53E-150	1.86E-145	1
RBM39	0.27588646	5.76E-110	1.94E-105	1
INSIG1	0.27496929	7.76E-104	2.62E-99	1
RBM8A	0.27403045	9.81E-128	3.31E-123	1
IL1RL1	0.27360029	6.00E-83	2.02E-78	1
DLC1	0.27337124	4.66E-110	1.57E-105	1

NNMT	0.27279509	3.12E-09	0.00010511	1
YBX3	0.27139718	5.41E-54	1.82E-49	1
RND1	0.27116017	1.19E-132	3.99E-128	1
LRRFIP1	0.27018673	6.67E-85	2.25E-80	1
MIR222HG	0.26986997	3.36E-82	1.13E-77	1
MAOA	0.26894162	5.18E-69	1.74E-64	1
RAPGEF5	0.26861143	9.92E-111	3.34E-106	1
NFKB2	0.26830933	1.31E-154	4.42E-150	1
NR4A1	0.26800635	1.42E-15	4.79E-11	1
MT-ND2	0.26733181	1.50E-12	5.07E-08	1
STOM	0.26595836	2.48E-46	8.36E-42	1
SDCBP	0.26592379	3.29E-74	1.11E-69	1
NCOA7	0.2645763	1.16E-105	3.92E-101	1
BRD2	0.26404424	3.74E-88	1.26E-83	1
ARC	0.26386915	2.71E-123	9.12E-119	1
HLA-DRB1	0.26322571	5.60E-47	1.89E-42	1
TGIF1	0.26144074	2.69E-99	9.05E-95	1
SELK	0.26122476	1.90E-94	6.40E-90	1
TRA2B	0.26076651	2.82E-91	9.52E-87	1
ARGLU1	0.2604307	1.21E-71	4.08E-67	1
RGS1	0.25975289	3.89E-64	1.31E-59	1
EIF1B	0.25792634	6.58E-59	2.22E-54	1
NFE2L2	0.25792606	5.12E-62	1.72E-57	1
SRSF2	0.25769667	1.53E-89	5.15E-85	1
THBD	0.25670672	2.24E-93	7.55E-89	1
TOP1	0.25577647	2.26E-77	7.61E-73	1
ARL4D	0.25433181	3.50E-17	1.18E-12	1
EMP1	0.25371148	0.00015496	1	1
PPP1R10	0.25315442	2.17E-84	7.32E-80	1
LACTB	0.25247594	2.86E-128	9.63E-124	1
FOSL2	0.25117989	6.36E-114	2.14E-109	1
ELF3	0.25116014	3.08E-71	1.04E-66	1
**PLA2G2A**	2.31113158	9.03E-54	3.04E-49	2
**MFAP5**	1.84107062	4.10E-68	1.38E-63	2
**SFRP2**	1.77915435	5.72E-53	1.93E-48	2
**SLPI**	1.65779807	9.52E-39	3.21E-34	2
**CFD**	1.52137547	7.26E-65	2.45E-60	2
**IGFBP6**	1.50802676	1.08E-63	3.65E-59	2
**C3**	1.27572745	1.65E-82	5.54E-78	2
**CCDC80**	1.26627051	5.75E-68	1.94E-63	2

**GSN**	1.23841183	1.60E-56	5.39E-52	2
**EFEMP1**	1.21500913	9.91E-77	3.34E-72	2
**CLU**	1.19240065	3.54E-58	1.19E-53	2
**SCARA5**	1.12033548	1.84E-81	6.19E-77	2
**SFRP4**	1.10595268	1.28E-19	4.31E-15	2
**PI16**	1.10252189	1.40E-63	4.73E-59	2
**PCOLCE2**	0.97719807	7.42E-63	2.50E-58	2
**CXCL14**	0.90894905	2.78E-31	9.38E-27	2
UAP1	0.90117706	2.35E-21	7.92E-17	2
FBLN1	0.88609573	2.41E-55	8.11E-51	2
APOD	0.8844281	1.29E-21	4.36E-17	2
MYOC	0.83938068	1.41E-15	4.74E-11	2
RARRES1	0.83886931	1.17E-15	3.95E-11	2
SFRP1	0.83853073	1.75E-65	5.91E-61	2
DCN	0.83460986	3.18E-64	1.07E-59	2
WISP2	0.77795869	2.52E-40	8.48E-36	2
HAS1	0.75931895	2.57E-58	8.67E-54	2
ADH1B	0.72123785	5.11E-34	1.72E-29	2
CST3	0.71562253	1.76E-62	5.93E-58	2
CPE	0.69402211	2.59E-46	8.74E-42	2
SERPINF1	0.69019128	3.02E-49	1.02E-44	2
HTRA3	0.68360971	2.02E-23	6.81E-19	2
MGST1	0.68084206	1.76E-58	5.93E-54	2
IGFBP7	0.67542159	1.52E-28	5.13E-24	2
PODN	0.66348036	1.18E-54	3.98E-50	2
C1R	0.65158676	3.98E-44	1.34E-39	2
C1QTNF3	0.63956369	9.32E-54	3.14E-49	2
RSPO3	0.63217311	7.43E-47	2.50E-42	2
FBLN2	0.62569861	2.57E-45	8.66E-41	2
IGF2	0.62278912	4.94E-56	1.66E-51	2
IGFBP5	0.61911704	2.37E-12	7.97E-08	2
C7	0.60874929	3.49E-13	1.17E-08	2
CADM3	0.60775943	1.46E-67	4.91E-63	2
PDGFRL	0.59348065	6.63E-57	2.23E-52	2
OGN	0.59130431	9.11E-36	3.07E-31	2
ITM2A	0.58756492	7.48E-36	2.52E-31	2
SRPX	0.54088454	8.04E-36	2.71E-31	2
MGP	0.5257938	2.20E-12	7.41E-08	2
SOD3	0.52555854	1.81E-39	6.09E-35	2
FGL2	0.49599369	6.19E-37	2.09E-32	2

AOX1	0.49232679	5.35E-65	1.80E-60	2
FSTL1	0.48727344	6.53E-24	2.20E-19	2
NUPR1	0.4805077	1.58E-29	5.31E-25	2
FBN1	0.47272032	9.82E-18	3.31E-13	2
CRYAB1	0.44777864	3.28E-13	1.11E-08	2
ABCA8	0.43980319	2.11E-34	7.11E-30	2
TXNIP	0.43873575	2.82E-16	9.51E-12	2
IFI27	0.43024447	8.19E-07	0.02759205	2
OMD	0.42459598	4.99E-42	1.68E-37	2
GAS1	0.42067367	1.51E-53	5.10E-49	2
GPNMB	0.41967712	5.82E-22	1.96E-17	2
SERPING1	0.41832162	1.15E-25	3.88E-21	2
AKAP12	0.41693227	4.31E-19	1.45E-14	2
PTGIS	0.41017804	1.81E-44	6.11E-40	2
CYBRD1	0.39758645	1.58E-43	5.32E-39	2
F10	0.39447835	1.31E-49	4.41E-45	2
ADIRF	0.39291168	2.26E-19	7.63E-15	2
CD55	0.38981469	3.77E-13	1.27E-08	2
C1S	0.38869057	6.20E-23	2.09E-18	2
IGFBP4	0.3800829	8.83E-13	2.97E-08	2
GALNT15	0.37519391	6.25E-35	2.11E-30	2
SCRG1	0.37227647	2.99E-11	1.01E-06	2
CHRDL1	0.36724768	8.12E-44	2.74E-39	2
C16orf89	0.36252409	1.32E-17	4.45E-13	2
MYC1	0.3608868	6.36E-15	2.14E-10	2
PLAC9	0.35601617	1.34E-16	4.51E-12	2
S100A4	0.35393108	3.61E-12	1.22E-07	2
KLF4	0.35340896	2.58E-09	8.71E-05	2
SLIT3	0.35022104	3.60E-25	1.21E-20	2
PLAT	0.3385448	3.74E-14	1.26E-09	2
PID1	0.33483271	5.07E-41	1.71E-36	2
MEDAG	0.33442809	1.71E-29	5.75E-25	2
CD34	0.32630086	1.08E-22	3.65E-18	2
LHFP	0.32038173	1.23E-18	4.14E-14	2
ITM2B	0.31922476	7.86E-23	2.65E-18	2
RHOB	0.31596885	1.38E-13	4.65E-09	2
PRSS23	0.31468644	4.11E-07	0.01384804	2
GPX31	0.3145912	3.40E-13	1.15E-08	2
CTSK	0.31221866	5.70E-15	1.92E-10	2
GNG11	0.30815124	1.25E-20	4.21E-16	2

CD59	0.30790657	4.19E-23	1.41E-18	2
CTSF	0.30691292	6.20E-21	2.09E-16	2
IGF1	0.30451617	2.82E-06	0.09501885	2
TSPAN8	0.30222144	2.03E-13	6.84E-09	2
CD248	0.29748534	1.49E-14	5.02E-10	2
PHGDH	0.2932529	1.11E-42	3.75E-38	2
FAM180B	0.29197351	6.27E-34	2.11E-29	2
F3	0.2872674	2.26E-14	7.60E-10	2
GPRC5A	0.28633573	6.17E-09	0.00020799	2
LEPR	0.28046248	3.21E-27	1.08E-22	2
MT1M1	0.27985174	3.86E-06	0.12990258	2
CILP	0.27922089	3.11E-23	1.05E-18	2
PRRX1	0.27843016	3.19E-25	1.07E-20	2
LRRN4CL	0.27814733	6.32E-47	2.13E-42	2
NFIA	0.27390102	1.04E-32	3.50E-28	2
PRELP1	0.27332831	8.60E-15	2.90E-10	2
TNXB	0.27294773	2.12E-15	7.16E-11	2
ADD3	0.27140974	4.04E-22	1.36E-17	2
BTG2	0.27063526	2.11E-08	0.00070933	2
LRP1	0.26385769	3.77E-24	1.27E-19	2
VIT	0.26298801	2.74E-50	9.24E-46	2
PROCR	0.26243272	8.89E-33	3.00E-28	2
FBLN5	0.26204632	5.93E-18	2.00E-13	2
CKB	0.26012304	3.07E-15	1.03E-10	2
GGT5	0.25939787	2.18E-31	7.33E-27	2
RTN4	0.25790425	9.60E-23	3.23E-18	2
EBF1	0.25760721	3.15E-42	1.06E-37	2
LGALS3BP	0.25735455	7.80E-15	2.63E-10	2
PLTP	0.25708178	2.65E-12	8.93E-08	2
AKR1C1	0.25695129	1.24E-19	4.18E-15	2
IFITM3	0.25487799	1.74E-11	5.86E-07	2
ANGPTL5	0.25278709	8.30E-33	2.80E-28	2
**COMP**	1.37546936	2.36E-138	7.97E-134	3
**ASPN**	1.20474859	6.91E-91	2.33E-86	3
**PENK**	1.03696441	1.47E-108	4.96E-104	3
**COL8A1**	1.01877441	4.04E-175	1.36E-170	3
**LTBP1**	0.85105941	1.05E-175	3.55E-171	3
**COL3A1**	0.80106364	9.75E-94	3.28E-89	3
**DPT**	0.7969568	2.66E-75	8.97E-71	3
**LTBP2**	0.79171426	1.40E-143	4.70E-139	3

**TSC22D1**	0.78099701	8.35E-82	2.81E-77	3
**MMP2**	0.77349981	3.02E-171	1.02E-166	3
**BGN**	0.75674066	1.45E-96	4.88E-92	3
**POSTN**	0.71544463	6.14E-67	2.07E-62	3
**MFAP4**	0.70076561	2.67E-106	9.00E-102	3
**A2M**	0.69456588	8.23E-65	2.77E-60	3
**MOXD1**	0.68476318	1.05E-140	3.52E-136	3
**VCAN**	0.68116192	1.59E-115	5.36E-111	3
SPARC	0.67402494	4.05E-84	1.37E-79	3
LUM	0.66273183	2.13E-88	7.16E-84	3
CES1	0.65285008	1.94E-81	6.52E-77	3
COL1A2	0.64711674	3.22E-95	1.08E-90	3
NBL1	0.63314831	2.59E-126	8.74E-122	3
ITIH5	0.63235684	1.77E-171	5.97E-167	3
COL6A3	0.62324101	5.62E-98	1.89E-93	3
ITGBL1	0.6091158	2.16E-84	7.27E-80	3
SPARCL1	0.60400918	6.36E-92	2.14E-87	3
DIO2	0.58350276	1.42E-137	4.80E-133	3
CCL13	0.58268694	2.54E-41	8.56E-37	3
COL14A1	0.58053292	7.69E-97	2.59E-92	3
PLPP1	0.57435929	5.59E-146	1.88E-141	3
SSPN	0.57380993	8.87E-145	2.99E-140	3
DKK3	0.56115058	7.20E-106	2.43E-101	3
ELN	0.56065061	1.98E-45	6.67E-41	3
PTGDS	0.54947229	1.71E-37	5.76E-33	3
IGFBP3	0.54778889	4.84E-48	1.63E-43	3
APCDD1	0.54388534	1.68E-129	5.66E-125	3
CFH	0.54078668	6.05E-62	2.04E-57	3
INHBA	0.53389836	2.60E-39	8.76E-35	3
EID1	0.52489847	3.83E-148	1.29E-143	3
SPON2	0.5166527	2.53E-50	8.54E-46	3
BMP5	0.51396461	1.98E-146	6.68E-142	3
ECM1	0.51251169	2.48E-121	8.35E-117	3
PLAC91	0.50724509	3.94E-78	1.33E-73	3
COL5A2	0.50607265	4.50E-105	1.52E-100	3
CDH11	0.49617314	4.42E-114	1.49E-109	3
IGFBP41	0.49345962	7.56E-57	2.55E-52	3
MFAP2	0.49223015	3.45E-129	1.16E-124	3
IGF11	0.49008591	8.12E-72	2.74E-67	3
LEPR1	0.48866548	7.88E-35	2.66E-30	3

THY1	0.48861807	3.82E-68	1.29E-63	3
TGFBI	0.48460942	6.65E-74	2.24E-69	3
ITGA8	0.48451182	4.23E-108	1.42E-103	3
SPON1	0.46927194	1.97E-156	6.64E-152	3
11-Sep	0.46375063	8.75E-146	2.95E-141	3
PPIC	0.45534875	4.14E-101	1.39E-96	3
CXCL141	0.4539239	6.14E-24	2.07E-19	3
ACTA2	0.45233389	2.44E-05	0.82368305	3
F2R	0.45167873	4.35E-45	1.47E-40	3
COL5A1	0.44834332	1.95E-85	6.56E-81	3
PLPP3	0.44722228	1.92E-39	6.47E-35	3
MGP1	0.44717267	1.39E-49	4.67E-45	3
COL6A2	0.43926399	6.72E-83	2.27E-78	3
MMP23B	0.43467264	5.24E-93	1.77E-88	3
TCF21	0.43432596	3.73E-49	1.26E-44	3
CAV1	0.43159439	3.61E-37	1.22E-32	3
COL15A1	0.43058021	5.15E-151	1.74E-146	3
COL1A1	0.42575339	1.08E-68	3.64E-64	3
FBLN21	0.42242181	2.26E-66	7.62E-62	3
MMP11	0.42138982	5.45E-87	1.84E-82	3
IGFBP51	0.41994805	6.73E-05	1	3
COL6A1	0.41984342	2.55E-93	8.59E-89	3
ANTXR1	0.4141106	8.66E-143	2.92E-138	3
OLFML3	0.41347629	2.37E-92	7.98E-88	3
OGN1	0.40955996	1.37E-74	4.61E-70	3
MAP1B	0.40737727	6.60E-74	2.22E-69	3
MXRA8	0.40729018	7.46E-99	2.51E-94	3
PCOLCE	0.4045321	1.78E-70	6.01E-66	3
TSPAN81	0.404335	4.63E-37	1.56E-32	3
CTHRC1	0.39927702	6.05E-57	2.04E-52	3
LMO4	0.39912529	4.07E-136	1.37E-131	3
FHL1	0.39673521	1.60E-47	5.38E-43	3
RCN3	0.39603018	2.34E-110	7.89E-106	3
TPPP3	0.3959484	8.52E-60	2.87E-55	3
TSC22D3	0.39573605	1.91E-46	6.45E-42	3
CLEC11A	0.39367014	4.47E-104	1.51E-99	3
ST3GAL4	0.39212984	2.87E-129	9.67E-125	3
ECM2	0.39147168	2.16E-153	7.28E-149	3
NDN	0.38641294	1.48E-124	4.97E-120	3
TAGLN	0.38620104	3.80E-08	0.00128085	3

EMILIN1	0.38091657	5.92E-58	2.00E-53	3
PLXDC2	0.38024483	2.04E-143	6.89E-139	3
P4HA2	0.37977831	4.01E-131	1.35E-126	3
GLT8D2	0.37505465	1.39E-111	4.67E-107	3
FBXO32	0.37405026	4.85E-84	1.64E-79	3
GUCY1A3	0.37403809	7.78E-131	2.62E-126	3
CTSK1	0.37140453	1.01E-58	3.41E-54	3
LSAMP	0.37082739	2.33E-117	7.85E-113	3
NUCKS1	0.36988296	1.11E-143	3.74E-139	3
PAPPA	0.36885065	5.69E-79	1.92E-74	3
HMCN1	0.3608555	2.07E-152	6.96E-148	3
ALDH2	0.35780462	9.20E-41	3.10E-36	3
GOLIM4	0.35497663	2.09E-139	7.04E-135	3
PTN	0.35408152	1.47E-99	4.95E-95	3
SERPINE2	0.35177658	4.66E-44	1.57E-39	3
MRC2	0.35131158	1.12E-130	3.77E-126	3
TMEM204	0.35008524	1.17E-85	3.94E-81	3
ZBTB20	0.34670476	5.17E-94	1.74E-89	3
NR2F1	0.34576149	1.43E-97	4.82E-93	3
MEOX2	0.34488611	1.26E-86	4.23E-82	3
TIMP2	0.34435526	3.76E-98	1.27E-93	3
CYGB	0.34317663	6.80E-103	2.29E-98	3
LOXL1	0.34221873	2.89E-80	9.75E-76	3
COL10A1	0.34131718	9.73E-147	3.28E-142	3
GPNMB1	0.3407451	5.30E-82	1.79E-77	3
LAMB1	0.33759256	1.02E-80	3.43E-76	3
STEAP1	0.33700375	1.12E-79	3.78E-75	3
EFEMP2	0.33536395	6.88E-87	2.32E-82	3
ANGPTL1	0.33497534	8.13E-107	2.74E-102	3
TMEM98	0.33341843	2.97E-109	9.99E-105	3
PLTP1	0.33341776	5.81E-65	1.96E-60	3
CPQ	0.33223725	8.18E-134	2.76E-129	3
LGALS3BP1	0.33007503	2.66E-79	8.95E-75	3
SSC5D	0.32741548	1.64E-156	5.52E-152	3
FBLN11	0.32677512	1.66E-46	5.59E-42	3
NGFRAP1	0.32378886	7.16E-85	2.41E-80	3
PEBP1	0.32291193	3.53E-127	1.19E-122	3
PDLIM3	0.32267069	6.96E-58	2.34E-53	3
ARL6IP5	0.32244234	7.93E-109	2.67E-104	3
SCPEP1	0.32195315	1.55E-111	5.24E-107	3

MYLK	0.31678879	2.27E-63	7.66E-59	3
TUBA1A	0.31669978	2.54E-27	8.57E-23	3
SFRP41	0.3166604	1.05E-28	3.53E-24	3
ZNF106	0.31653943	2.46E-90	8.29E-86	3
PALLD	0.31610416	2.90E-85	9.78E-81	3
FMO3	0.31591833	1.14E-58	3.84E-54	3
MYL9	0.31583732	1.68E-33	5.65E-29	3
CERCAM	0.31437749	1.53E-83	5.15E-79	3
LIMCH1	0.31422816	8.74E-48	2.95E-43	3
USP18	0.31418235	8.67E-155	2.92E-150	3
TNFSF13B	0.31385925	5.94E-48	2.00E-43	3
SPRY1	0.31329674	2.34E-44	7.88E-40	3
CD81	0.31196949	2.79E-96	9.41E-92	3
CLMP	0.31065367	1.23E-118	4.15E-114	3
FBN11	0.31040931	5.31E-76	1.79E-71	3
LAPTM4A	0.30991314	2.54E-64	8.56E-60	3
CALD1	0.30967835	2.57E-42	8.66E-38	3
ANGPTL2	0.30950862	3.38E-88	1.14E-83	3
ITM2B1	0.30540928	5.49E-61	1.85E-56	3
CALU	0.30534989	4.51E-110	1.52E-105	3
MDK	0.30490493	2.33E-69	7.84E-65	3
C1QTNF2	0.30427513	3.11E-128	1.05E-123	3
ROBO2	0.30397113	5.37E-125	1.81E-120	3
PLEKHA5	0.3030438	4.22E-109	1.42E-104	3
LRRC17	0.3027368	1.16E-91	3.89E-87	3
NKD2	0.30192046	5.33E-74	1.80E-69	3
FHL2	0.30122191	3.86E-90	1.30E-85	3
CXCL12	0.3002285	6.54E-51	2.20E-46	3
RUNX1T1	0.29985516	3.47E-118	1.17E-113	3
RAB34	0.29955585	2.91E-116	9.82E-112	3
AEBP1	0.2961943	8.70E-34	2.93E-29	3
SAMHD1	0.29491251	4.94E-63	1.67E-58	3
PMP22	0.29485104	7.93E-39	2.67E-34	3
ANXA6	0.29466759	5.22E-104	1.76E-99	3
C1S1	0.29089894	1.04E-40	3.50E-36	3
7-Sep	0.29031115	4.68E-113	1.58E-108	3
ANGPT1	0.29027965	2.35E-55	7.92E-51	3
APP	0.289141	4.21E-78	1.42E-73	3
TMEM119	0.28605384	2.85E-101	9.60E-97	3
FZD1	0.28604302	5.51E-100	1.86E-95	3

PIK3R1	0.28590581	7.88E-72	2.66E-67	3
LAMA2	0.28515716	6.43E-57	2.17E-52	3
CD9	0.2834372	7.81E-20	2.63E-15	3
ENC1	0.28297047	9.56E-118	3.22E-113	3
PSAP	0.28172855	3.08E-82	1.04E-77	3
SDC2	0.28029875	2.20E-32	7.42E-28	3
RHOBTB3	0.27932853	1.12E-75	3.78E-71	3
CHN1	0.2773863	3.30E-92	1.11E-87	3
GDF10	0.27702581	1.14E-51	3.83E-47	3
HTRA1	0.27676311	2.96E-59	9.96E-55	3
SERPINF11	0.27654186	1.41E-37	4.75E-33	3
NFIX	0.27585416	1.04E-126	3.51E-122	3
TMEM100	0.27542843	1.08E-15	3.64E-11	3
LTBP3	0.2749463	2.75E-126	9.25E-122	3
PLEKHH2	0.27416062	4.20E-44	1.42E-39	3
LAMP5	0.27313763	1.84E-141	6.19E-137	3
TTC3	0.2718449	2.97E-96	1.00E-91	3
FN1	0.27180637	2.42E-39	8.15E-35	3
RBMS3	0.27155719	3.24E-84	1.09E-79	3
PAM	0.26927847	4.87E-120	1.64E-115	3
LAMP1	0.26828939	2.00E-101	6.74E-97	3
FSTL11	0.26788322	1.08E-47	3.64E-43	3
BICC1	0.26712775	1.17E-102	3.95E-98	3
RERG	0.26693626	1.14E-95	3.83E-91	3
PMEPA1	0.26626676	6.69E-109	2.25E-104	3
TCEAL7	0.26601608	3.37E-106	1.14E-101	3
PLXDC1	0.26584621	1.33E-123	4.48E-119	3
CAPZB	0.26545113	1.24E-113	4.17E-109	3
C1QTNF7	0.26526723	1.02E-113	3.43E-109	3
TNXB1	0.26278891	6.16E-25	2.07E-20	3
PRG4	0.26255619	3.70E-65	1.25E-60	3
BMP4	0.26254228	6.97E-84	2.35E-79	3
FKBP7	0.26190399	2.36E-105	7.96E-101	3
FAM114A1	0.26078088	4.94E-100	1.67E-95	3
GPX8	0.26028524	2.85E-81	9.60E-77	3
MAGED2	0.25995343	3.25E-103	1.09E-98	3
ENAH	0.25867907	4.33E-83	1.46E-78	3
LEPROT	0.25828747	1.72E-75	5.80E-71	3
ITM2C	0.25745452	8.09E-78	2.73E-73	3
MXRA5	0.25657413	2.85E-103	9.61E-99	3

TMEM45A	0.2562141	9.26E-104	3.12E-99	3
CST1	0.25608478	6.02E-44	2.03E-39	3
PRDX2	0.25599201	4.88E-92	1.65E-87	3
OSR2	0.25533126	5.99E-64	2.02E-59	3
PDZRN3	0.25460258	7.35E-161	2.48E-156	3
DPYSL3	0.25451616	3.89E-85	1.31E-80	3
EPHX1	0.25364004	1.96E-61	6.59E-57	3
ATP6AP2	0.25179988	1.20E-106	4.03E-102	3
CTSF1	0.2513371	7.60E-82	2.56E-77	3
TSPAN13	0.25132961	1.73E-78	5.82E-74	3
FKBP10	0.25090697	5.64E-73	1.90E-68	3
JADE1	0.25068187	2.51E-48	8.46E-44	3
SH3BGRL	0.25037376	3.34E-107	1.12E-102	3
**COL1A11**	1.29844505	1.49E-51	5.02E-47	4
**TIMP1**	1.20696335	2.24E-46	7.54E-42	4
**CTHRC11**	1.19785846	4.62E-51	1.56E-46	4
**LGALS1**	0.94428925	8.05E-78	2.71E-73	4
**ISLR**	0.92833726	4.52E-41	1.52E-36	4
**CRABP2**	0.89380691	1.86E-25	6.28E-21	4
**SH3BGRL3**	0.87993533	8.54E-64	2.88E-59	4
**TNC**	0.87359963	4.46E-24	1.50E-19	4
**POSTN1**	0.8630085	1.35E-30	4.56E-26	4
**TPM2**	0.84250308	3.14E-39	1.06E-34	4
**FN11**	0.8215858	1.14E-19	3.85E-15	4
**SPARC1**	0.8071698	5.02E-39	1.69E-34	4
**S100A16**	0.80470324	9.83E-61	3.31E-56	4
**COL3A11**	0.78367491	7.33E-35	2.47E-30	4
**TUBA1B**	0.73733309	2.91E-09	9.82E-05	4
**COL1A21**	0.71075013	1.49E-31	5.02E-27	4
IGFL2	0.68778325	5.82E-35	1.96E-30	4
TUBB	0.67329056	2.09E-55	7.04E-51	4
RP11-400N13.3	0.67186789	4.59E-45	1.55E-40	4
RPL22L1	0.6672537	6.36E-61	2.14E-56	4
MYL91	0.6625687	2.57E-22	8.66E-18	4
PFN1	0.66071242	2.78E-66	9.38E-62	4
SULF1	0.61894377	6.58E-33	2.22E-28	4
TPM4	0.60834844	2.71E-61	9.14E-57	4
GAPDH	0.60218613	3.73E-62	1.26E-57	4
PKM	0.60100998	8.09E-51	2.73E-46	4

MXRA51	0.60081368	2.47E-33	8.31E-29	4
PDLIM4	0.57363731	5.47E-56	1.84E-51	4
CERCAM1	0.57339347	7.79E-32	2.62E-27	4
P4HB	0.56972356	9.95E-56	3.35E-51	4
KDELR3	0.56722476	7.44E-40	2.51E-35	4
LOXL11	0.5559075	3.65E-39	1.23E-34	4
COL5A21	0.54293287	1.51E-28	5.09E-24	4
RPS19	0.54280805	8.65E-62	2.91E-57	4
MDK1	0.54266528	3.47E-24	1.17E-19	4
RPS18	0.53733205	2.38E-67	8.03E-63	4
TMSB10	0.53688914	7.07E-63	2.38E-58	4
SERPINE1	0.53390327	1.19E-18	4.00E-14	4
RPL28	0.53117169	1.06E-75	3.59E-71	4
S100A10	0.53024548	5.30E-32	1.79E-27	4
KDELR2	0.52993359	2.50E-57	8.41E-53	4
TAGLN1	0.5185172	1.84E-07	0.00620004	4
VIM	0.51505608	1.50E-40	5.05E-36	4
COL5A11	0.51148438	5.10E-31	1.72E-26	4
ANXA2	0.50827359	2.90E-30	9.79E-26	4
COL6A11	0.50567618	6.82E-20	2.30E-15	4
LY6E	0.49156986	1.68E-51	5.64E-47	4
ADAM12	0.48636955	5.08E-38	1.71E-33	4
IGFBP2	0.48480091	2.03E-14	6.83E-10	4
RPS2	0.47970487	6.65E-57	2.24E-52	4
SERF2	0.47691659	1.78E-53	6.01E-49	4
ACTB	0.47145348	1.26E-24	4.24E-20	4
LRRC171	0.47116928	1.51E-33	5.07E-29	4
RAB13	0.46726407	5.73E-61	1.93E-56	4
RPSA	0.46383756	9.69E-59	3.26E-54	4
ISG15	0.46354687	2.05E-07	0.00690455	4
CKAP4	0.46207419	1.48E-62	4.98E-58	4
NME1	0.46199302	4.09E-60	1.38E-55	4
ARPC1B	0.46142237	5.39E-69	1.82E-64	4
ACTA21	0.45983593	1.04E-07	0.00348772	4
EIF5A	0.45636617	1.00E-31	3.39E-27	4
IL32	0.45152785	5.38E-20	1.81E-15	4
YBX1	0.44885056	2.08E-63	7.02E-59	4
TMSB4X	0.44710546	1.09E-27	3.68E-23	4
FKBP101	0.44644192	9.05E-34	3.05E-29	4
PRKCDBP	0.44516244	2.09E-29	7.05E-25	4

EEF1B2	0.44147067	1.46E-64	4.93E-60	4
S100A6	0.44083209	1.61E-18	5.43E-14	4
OST4	0.4405114	1.95E-67	6.55E-63	4
TMED3	0.44020114	4.13E-58	1.39E-53	4
VKORC1	0.43842735	7.02E-40	2.36E-35	4
S100A11	0.4374615	3.74E-35	1.26E-30	4
RPL18A	0.43540376	7.84E-64	2.64E-59	4
RCN1	0.43313659	3.30E-43	1.11E-38	4
ACTG1	0.43157312	1.70E-32	5.71E-28	4
RPL12	0.43082721	1.41E-56	4.74E-52	4
RPS12	0.42981082	3.75E-50	1.27E-45	4
RPL4	0.42933164	1.93E-58	6.49E-54	4
RPS17	0.42633846	2.98E-58	1.00E-53	4
SRM	0.4251288	3.65E-64	1.23E-59	4
AP2S1	0.42404162	1.13E-69	3.81E-65	4
ARPC2	0.42343053	1.10E-53	3.71E-49	4
COL6A31	0.42309412	2.48E-19	8.36E-15	4
ACTG2	0.42120217	0.00356516	1	4
RPS3	0.42060428	1.36E-53	4.57E-49	4
BGN1	0.42017526	1.47E-18	4.96E-14	4
RPS26	0.41971596	4.01E-39	1.35E-34	4
SERPINH1	0.41835486	1.84E-28	6.19E-24	4
MEDAG1	0.41472273	3.03E-44	1.02E-39	4
RPL29	0.40998353	4.54E-58	1.53E-53	4
SEC61G	0.40955005	2.66E-46	8.97E-42	4
TUBA1C	0.40876363	2.23E-19	7.50E-15	4
GAP43	0.40531549	2.34E-46	7.88E-42	4
TPM1	0.40361484	8.40E-14	2.83E-09	4
RPL36	0.4029367	2.64E-51	8.91E-47	4
RPL27	0.40172483	3.17E-74	1.07E-69	4
RPL41	0.39796936	1.89E-59	6.35E-55	4
RPLP0	0.39750107	3.28E-43	1.11E-38	4
RPL37	0.39580768	2.17E-52	7.31E-48	4
RHOC	0.39300021	4.30E-49	1.45E-44	4
RPL7A	0.39271166	2.03E-49	6.84E-45	4
RPS4X	0.38773104	8.13E-35	2.74E-30	4
SLC12A8	0.38758725	1.44E-47	4.86E-43	4
EMP3	0.38522209	6.82E-13	2.30E-08	4
RPL8	0.3841066	1.61E-61	5.43E-57	4
RPS24	0.38113771	1.11E-55	3.74E-51	4

FKBP11	0.38105649	4.68E-38	1.58E-33	4
THY11	0.38105283	8.31E-21	2.80E-16	4
FKBP1A	0.38033669	3.16E-56	1.07E-51	4
RPS5	0.37800244	1.74E-50	5.85E-46	4
PRDX4	0.37797368	2.73E-45	9.20E-41	4
OSTC	0.3776751	4.39E-56	1.48E-51	4
RPL35	0.37556482	6.20E-53	2.09E-48	4
RPL10A	0.37505066	9.55E-48	3.22E-43	4
RPL37A	0.37502107	2.92E-48	9.82E-44	4
PDPN	0.37475298	9.78E-42	3.30E-37	4
PPIB	0.3732019	1.30E-35	4.38E-31	4
RPL27A	0.37089741	1.89E-54	6.37E-50	4
C9orf16	0.36822703	4.64E-52	1.56E-47	4
ENO1	0.3648622	9.13E-11	3.08E-06	4
PDLIM7	0.3630391	6.09E-34	2.05E-29	4
CHPF	0.36024928	8.98E-27	3.02E-22	4
RPLP1	0.35958731	6.76E-55	2.28E-50	4
PHPT1	0.35854721	2.86E-40	9.63E-36	4
TGFBI1	0.35852971	8.05E-24	2.71E-19	4
RPS7	0.35555465	1.02E-57	3.44E-53	4
ATP5E	0.35459659	5.23E-56	1.76E-51	4
YIF1A	0.35398324	6.62E-49	2.23E-44	4
PGAM1	0.35263119	1.96E-51	6.62E-47	4
RCN31	0.35047247	4.46E-25	1.50E-20	4
RPS6	0.35029303	5.09E-42	1.72E-37	4
CFL1	0.34983903	1.63E-39	5.48E-35	4
UQCRH	0.34796152	1.47E-75	4.94E-71	4
PYCR1	0.34728811	4.53E-44	1.53E-39	4
ALDH1A3	0.34611501	1.14E-28	3.85E-24	4
PPIC1	0.3419434	1.95E-21	6.57E-17	4
PRAF2	0.3417437	1.00E-47	3.39E-43	4
THBS2	0.34166097	3.87E-27	1.30E-22	4
GUK1	0.34154632	9.63E-60	3.24E-55	4
TCEB2	0.33974566	3.74E-57	1.26E-52	4
PTMS	0.33835659	5.03E-38	1.69E-33	4
SSR4	0.33720162	4.01E-38	1.35E-33	4
TPPP31	0.33681742	0.00475518	1	4
SEC61B	0.33622503	8.09E-50	2.73E-45	4
RPL13	0.33222258	1.51E-41	5.09E-37	4
GNB2L1	0.33216236	4.24E-43	1.43E-38	4

DPYSL31	0.33182561	8.27E-28	2.79E-23	4
RPL39	0.3316017	2.98E-38	1.00E-33	4
RPL32	0.33159256	1.04E-42	3.51E-38	4
RPL6	0.32863541	6.87E-43	2.32E-38	4
PRELID1	0.32856789	5.24E-66	1.77E-61	4
UQCRQ	0.32695477	2.39E-56	8.05E-52	4
RPS15A	0.32670429	6.49E-42	2.19E-37	4
RPS14	0.32204961	3.29E-44	1.11E-39	4
MYDGF	0.32134103	1.44E-35	4.86E-31	4
HMGN2	0.3210372	1.29E-17	4.36E-13	4
NDUFS5	0.31767904	5.61E-61	1.89E-56	4
CD99	0.31716275	1.01E-25	3.41E-21	4
UBA52	0.31627582	8.63E-61	2.91E-56	4
NDUFB2	0.31458815	9.49E-53	3.20E-48	4
RPL15	0.31398325	7.16E-44	2.41E-39	4
ATOX1	0.31198777	7.85E-59	2.64E-54	4
RPS3A	0.31151348	3.71E-36	1.25E-31	4
C12orf75	0.31038521	5.61E-27	1.89E-22	4
RPL3	0.30985103	2.32E-29	7.81E-25	4
UCHL1	0.30842806	1.24E-33	4.18E-29	4
MINOS1	0.30795236	6.40E-52	2.16E-47	4
RPL19	0.30761989	1.35E-47	4.56E-43	4
SLC25A6	0.30713931	2.08E-44	7.00E-40	4
RPLP2	0.30621181	3.72E-43	1.25E-38	4
RPS21	0.30539039	5.40E-39	1.82E-34	4
MYL6	0.30529078	2.54E-21	8.57E-17	4
RBP1	0.30526845	2.56E-21	8.62E-17	4
CLEC11A1	0.30501779	1.17E-22	3.96E-18	4
PLP2	0.30401898	2.03E-21	6.86E-17	4
TYMS	0.30248585	3.48E-20	1.17E-15	4
ALDOA	0.30187222	4.37E-47	1.47E-42	4
RPS10	0.29939209	3.91E-48	1.32E-43	4
RPS29	0.29875795	6.81E-36	2.29E-31	4
RPS15	0.29853655	2.59E-44	8.71E-40	4
TSPO	0.29830292	1.77E-29	5.98E-25	4
RPL23	0.29790666	3.87E-41	1.30E-36	4
IFI27L2	0.2960139	2.87E-44	9.66E-40	4
RPS8	0.29588297	7.01E-31	2.36E-26	4
PPP1CC	0.29512341	3.71E-40	1.25E-35	4
PDLIM2	0.29490457	5.23E-33	1.76E-28	4

RPL18	0.29324552	1.35E-38	4.56E-34	4
RBM3	0.29180076	1.10E-50	3.70E-46	4
RANBP1	0.29124028	8.83E-42	2.97E-37	4
TSPAN2	0.28917244	4.02E-42	1.36E-37	4
AP2M1	0.28782722	7.62E-48	2.57E-43	4
COX8A	0.28759022	4.05E-55	1.36E-50	4
APRT	0.28711977	2.47E-69	8.32E-65	4
EEF2	0.28704439	3.53E-28	1.19E-23	4
SDC21	0.28687419	3.56E-10	1.20E-05	4
RPS11	0.28548841	5.51E-46	1.86E-41	4
ISOC2	0.28539479	3.64E-55	1.23E-50	4
RPL26	0.28521882	5.54E-30	1.87E-25	4
RPL7	0.28406287	2.61E-28	8.79E-24	4
RPL13A	0.28373432	5.03E-30	1.69E-25	4
CD2481	0.28361733	3.53E-24	1.19E-19	4
ANAPC11	0.28326632	1.17E-60	3.94E-56	4
SH3PXD2A	0.28183214	3.98E-24	1.34E-19	4
EEF1A1	0.28118629	1.62E-23	5.45E-19	4
SSR3	0.28085731	2.28E-55	7.69E-51	4
RAMP1	0.28063436	1.05E-12	3.52E-08	4
RPL14	0.28056359	3.15E-32	1.06E-27	4
SPATS2L	0.27552316	4.54E-45	1.53E-40	4
MIF	0.27397043	3.52E-43	1.19E-38	4
FAP	0.27387197	2.26E-25	7.61E-21	4
RPS28	0.27282035	6.24E-28	2.10E-23	4
NME4	0.27097092	4.96E-47	1.67E-42	4
RPL5	0.27060993	5.59E-25	1.88E-20	4
IFI271	0.26998631	0.00045364	1	4
GTF3A	0.2699861	8.62E-64	2.90E-59	4
GPC1	0.26919033	2.15E-49	7.25E-45	4
CALU1	0.26888378	9.40E-28	3.17E-23	4
RAN	0.2682272	1.72E-24	5.78E-20	4
HDLBP	0.26685978	6.00E-39	2.02E-34	4
GSTP1	0.26646919	2.20E-20	7.43E-16	4
SSR2	0.26568697	1.33E-48	4.47E-44	4
C19orf53	0.26483295	1.27E-80	4.27E-76	4
NDUFA4	0.26394317	8.54E-36	2.88E-31	4
RHOA	0.26331747	3.25E-33	1.09E-28	4
RPL11	0.26242144	6.28E-30	2.12E-25	4
TPI1	0.26141372	1.09E-22	3.66E-18	4

RPL30	0.26135426	4.09E-33	1.38E-28	4
KIAA0101	0.2609236	0.00120446	1	4
HINT1	0.26058066	1.66E-54	5.58E-50	4
GDI2	0.26004092	3.20E-52	1.08E-47	4
FSCN1	0.25937837	2.66E-41	8.98E-37	4
GPX81	0.25924628	2.51E-22	8.45E-18	4
HMGN1	0.257853	7.38E-39	2.49E-34	4
ATP5D	0.2576277	5.66E-68	1.91E-63	4
PSME2	0.25718662	8.65E-25	2.92E-20	4
MARCKSL1	0.25580118	1.37E-17	4.62E-13	4
TMEM167A	0.25569589	9.71E-46	3.27E-41	4
JTB	0.25526618	5.70E-63	1.92E-58	4
HCFC1R1	0.25525054	8.00E-36	2.70E-31	4
TMEM230	0.25484905	1.37E-34	4.62E-30	4
TPT1	0.25478134	1.15E-18	3.89E-14	4
ATP5G1	0.25459971	2.08E-56	7.00E-52	4
RPL38	0.25457332	6.18E-32	2.08E-27	4
RPS20	0.25357129	3.27E-30	1.10E-25	4
ETHE1	0.25323922	7.08E-56	2.38E-51	4
RPL36A	0.25198007	4.54E-34	1.53E-29	4
C1orf122	0.25185452	1.69E-34	5.69E-30	4
NPM1	0.25123509	6.70E-24	2.26E-19	4
RPL24	0.25088454	2.01E-34	6.78E-30	4

Supplementary Table S4. List of genes related to inflammation and fibrosis with references.

	GeneSymbol	Gene Name	REF
Fibroblasts Cluster-1	ICAM1	Intercellular Adhesion Molecule 1	(1)
	CXCL1	C-X-C Motif Chemokine Ligand 1	(2)
	CXCL2	C-X-C Motif Chemokine Ligand 2	(2)
	CXCL3	C-X-C Motif Chemokine Ligand 3	(2)
	CXCL8	C-X-C Motif Chemokine Ligand 8	(2)
	CCL2	C-C Motif Chemokine Ligand 2	(2)
	IL6	Interleukin 6	(3)
	PTX3	Pentraxin 3	(4)
	IER3	Immediate Early Response 3	(5)
	GADD45B	Growth Arrest And DNA Damage Inducible Beta	(6)
	THBS1	Thrombospondin 1	(7)
	SOD2	Superoxide Dismutase 2	(8)
	NFKBIA	NFKB Inhibitor Alpha	(9)
Fibroblasts Cluster-2	CXCL14	C-X-C Motif Chemokine Ligand 14	(10)
	SFRP2	Secreted Frizzled Related Protein 2	(11)
	SFRP4	Secreted Frizzled Related Protein 4	(11)
Fibroblasts Cluster-3	POSTN	Periostin	(12)
	ASPN	Asporin	(13)
	LTBP2	Latent Transforming Growth Factor Beta Binding Protein 2	(14)
	LTBP1	Latent Transforming Growth Factor Beta Binding Protein 1	(15)
	BGN	Biglycan	(16)
	DPT	Dermatopontin	(17, 18)
	COL3A1	Collagen Type III Alpha 1 Chain	(19)
	COL8A1	Collagen Type VIII Alpha 1 Chain	(12)
	MFAP4	Microfibril Associated Protein 4	(18)
	VCAM	Vascular Cell Adhesion Molecule 1	(20)
	COMP	Cartilage Oligomeric Matrix Protein	(21)
	MMP2	Matrix Metallopeptidase 2	(22)
Fibroblasts Cluster-4	TNC	Tenascin C	(23)
	SPARC	Secreted Protein Acidic And Cysteine Rich	(24-26)
	POSTN	Periostin	(12)
	FN1	Fibronectin 1	(27)
	TPM2	Tropomyosin 2	(28)

Supplementary Table S5. Ribosomal protein mRNAs are globally downregulated in C2G2 compared to C2G1 cells in Cluster-2.

**GeneSymbol**	**avg_logFC**	**p_val**	**p_val_adj**
RPS27	0.52	5.91E-13	1.99E-08
RPS28	0.45	2.61E-10	8.79E-06
RPS18	0.45	7.25E-11	2.44E-06
RPS2	0.45	1.87E-09	6.31E-05
RPLP0	0.44	2.22E-07	7.47E-03
RPS29	0.43	7.39E-08	2.49E-03
RPL34	0.42	1.51E-12	5.09E-08
RPSA	0.42	5.22E-10	1.76E-05
RPL31	0.42	4.50E-12	1.52E-07
RPL32	0.42	1.91E-12	6.45E-08
RPS12	0.42	3.77E-08	1.27E-03
RPL18A	0.42	2.43E-11	8.19E-07
RPS8	0.41	3.99E-10	1.34E-05
RPS5	0.41	2.11E-10	7.09E-06
RPS19	0.41	3.83E-08	1.29E-03
RPS14	0.40	1.07E-12	3.60E-08
RPL7	0.40	4.93E-10	1.66E-05
RPL39	0.40	4.74E-10	1.60E-05
RPL13	0.39	4.38E-11	1.48E-06
RPL41	0.39	2.82E-10	9.50E-06
RPS6	0.39	9.39E-10	3.16E-05
RPL11	0.39	7.46E-12	2.51E-07
RPL37A	0.39	2.25E-09	7.59E-05
RPL18	0.39	6.82E-11	2.30E-06
RPS3	0.39	2.56E-09	8.64E-05
RPL13A	0.39	5.63E-10	1.90E-05
RPLP2	0.38	1.16E-11	3.90E-07
RPL10A	0.38	3.35E-10	1.13E-05
RPL37	0.38	4.85E-11	1.63E-06
RPL36	0.38	6.35E-09	2.14E-04
RPL35A	0.38	2.76E-12	9.29E-08
RPL26	0.38	5.70E-11	1.92E-06
RPL3	0.37	1.68E-09	5.65E-05
RPL10	0.37	9.58E-11	3.23E-06
RPS25	0.37	3.27E-12	1.10E-07
RPL21	0.37	4.30E-11	1.45E-06
RPL5	0.37	2.81E-09	9.46E-05
RPS15A	0.37	2.71E-10	9.14E-06
RPL23A	0.37	8.01E-11	2.70E-06
RPS3A	0.36	2.38E-09	8.02E-05

RPL23	0.36	4.85E-11	1.63E-06
RPS23	0.36	7.21E-10	2.43E-05
RPL27A	0.36	1.84E-10	6.19E-06
RPL35	0.36	6.08E-10	2.05E-05
RPS16	0.35	3.17E-10	1.07E-05
RPL30	0.35	2.29E-11	7.70E-07
RPL14	0.35	1.65E-09	5.55E-05
RPL38	0.35	1.42E-11	4.80E-07
RPL29	0.35	2.51E-10	8.45E-06
RPS20	0.34	1.29E-10	4.34E-06
RPL28	0.34	9.03E-08	3.04E-03
RPS27A	0.34	1.56E-09	5.24E-05
RPL7A	0.34	9.06E-09	3.05E-04
RPS13	0.34	7.25E-11	2.44E-06
RPLP1	0.34	4.39E-10	1.48E-05
RPL4	0.34	3.90E-09	1.31E-04
RPS24	0.34	1.80E-08	6.06E-04
RPS17	0.34	8.59E-09	2.90E-04
RPL6	0.33	5.91E-09	1.99E-04
RPS7	0.33	6.23E-09	2.10E-04
RPL12	0.33	9.73E-09	3.28E-04
RPS21	0.32	9.06E-09	3.05E-04
RPL9	0.32	1.65E-09	5.55E-05
RPL8	0.32	1.18E-09	3.96E-05
RPS15	0.32	3.13E-09	1.06E-04
RPL19	0.32	2.02E-10	6.82E-06
RPL15	0.32	4.15E-10	1.40E-05
RPS9	0.30	5.29E-08	1.78E-03
RPL27	0.29	1.20E-09	4.04E-05
RPS11	0.27	7.19E-09	2.42E-04
RPL22	0.27	8.29E-09	2.79E-04
RPL36A	0.27	4.27E-09	1.44E-04
RPL24	0.27	3.02E-09	1.02E-04
RPS10	0.26	3.37E-09	1.14E-04

Supplementary Table S6A. Differential gene expression for copper-binding genes between Cluster 1 (normal) and Cluster 3 (fibrotic) cells. Part I: upregulated genes in Cluster 3; part II: downregulated genes in Cluster 3; part III: genes not differentially expressed.

GeneSymbol	avg_logFC	p_val	p_val_adj
SPARC	-1.3377614	6.38E-129	8.29E-128
MOXD1	-0.805859	5.14E-138	8.92E-137
LOXL1	-0.6321814	3.42E-147	1.78E-145
APP	-0.3934383	7.56E-93	3.93E-92
S100A13	-0.3868142	3.37E-79	1.35E-78
PAM	-0.3627751	3.22E-145	8.38E-144
PARK7	-0.2816728	1.78E-127	1.85E-126
MT-CO1	-0.2427346	9.02E-20	1.30E-19
HEPH	-0.2400801	1.34E-126	1.16E-125
CCS	-0.2338949	2.75E-124	2.05E-123
LOX	-0.1978914	1.61E-55	3.64E-55
PRNP	-0.1937362	9.28E-49	2.01E-48
SOD1	-0.1786077	1.04E-47	2.00E-47
ATOX1	-0.1583605	4.79E-48	9.57E-48
COX11	-0.126048	1.46E-106	9.46E-106
CUTA	-0.1195893	1.18E-37	2.11E-37
GPC1	-0.1080996	2.76E-67	7.54E-67
LOXL2	-0.0680043	2.78E-70	8.03E-70
COMMD1	-0.0600248	1.34E-48	2.79E-48
F5	-0.0380839	4.23E-67	1.10E-66
CUTC	-0.0373513	1.44E-10	1.79E-10
LOXL3	-0.0172311	8.70E-72	2.66E-71
SCO1	-0.0131812	5.80E-19	8.15E-19
CP	-0.0130793	6.53E-11	8.28E-11
AFP	-0.010591	8.69E-09	1.04E-08
SLC31A1	-0.010361	9.00E-14	1.20E-13
S100B	-0.0092444	2.24E-24	3.53E-24
TYRP1	-0.0089608	1.68E-12	2.19E-12
ENOX1	-0.0085989	4.63E-78	1.72E-77
ATP7B	-0.0073743	1.18E-60	2.78E-60
LOXL4	-0.0054964	3.23E-75	1.12E-74
ENOX2	-0.0044788	7.96E-21	1.18E-20
ATP7A	-0.0010764	8.83E-09	1.04E-08
TYR	-0.0008232	3.62E-32	6.07E-32
DBH	-0.0006459	4.58E-41	8.51E-41

**ALB**	-0.0005889	1.87E-33	3.25E-33
**AOC1**	-0.0004768	0.00286679	0.00324071
**S100A5**	-0.0004363	1.53E-05	1.77E-05
**MEMO1**	-4.96E-05	0.04242307	0.0450204
**HEPHL1**	0.00017238	1.18E-23	1.81E-23
**AOC2**	0.00621501	4.38E-17	5.99E-17
**S100A12**	0.02496471	6.04E-106	3.49E-105
**SOD3**	0.06287363	0.01147174	0.01269214
**SCO2**	0.0657051	6.48E-80	3.06E-79
**SLC31A2**	0.0680623	1.03E-62	2.55E-62
**MT-CO2**	0.08048577	0.01913214	0.02072649
**MAP2K1**	0.08635951	7.15E-72	2.32E-71
**COX17**	0.09373701	1.24E-25	2.01E-25
**SNCA**	0.13475549	1.71E-79	7.41E-79
MT3	0.00097683	0.13337185	0.13870672
LTF	0.00167063	0.44786085	0.44786085
AOC3	0.04512818	0.40558582	0.41353848
MT4	0	NA	NA

Supplementary Table S6B. Differential gene expression for copper-binding genes between Cluster 2 C2G1 (velocity towards normal SS1) and C2G2 (velocity towards fibrotic SS2). Part I: upregulated genes in C2G2; part II: downregulated genes in C2G2; part III: genes not differentially expressed.

**GeneSymbol**	**avg_FC**	**P_value**	**P.adj**
**SPARC**	-0.5614634	2.76E-05	0.00035846
**MOXD1**	-0.3481906	7.33E-06	0.00012698
**PAM**	-0.2663576	1.11E-06	5.80E-05
**APP**	-0.2206723	0.00069975	0.0045484
**MT-CO1**	-0.2196486	0.00877742	0.02535698
**LOXL1**	-0.1920861	0.00139077	0.00657453
**HEPH**	-0.1615404	4.83E-06	0.00012555
**MT-CO2**	-0.1596061	0.01862497	0.04524043
**PRNP**	-0.1549761	0.00196865	0.00853082
**CCS**	-0.1272404	0.00111046	0.00577442
**SLC31A1**	-0.0229862	0.00024172	0.00179562
**LOXL2**	-0.0229013	0.01914018	0.04524043
**TYRP1**	-0.020903	0.00413171	0.01432328
**ENOX1**	-0.0162381	0.00013166	0.00114103
**LOXL3**	-0.0137429	0.0048972	0.01591591
**SCO1**	-0.0123905	0.01779322	0.04524043
**LTF**	-0.0058866	0.00084124	0.00486051
**ATP7B**	-0.0029025	0.00641786	0.01963111
**LOXL4**	-0.0027467	0.00288503	0.0115401
**MT3**	-0.0019358	5.34E-05	0.00055549
**SNCA**	0.05013874	0.01397206	0.03823934
**S100A12**	0.00212072	0.00371018	0.01378066
SOD3	-0.1361267	0.17589636	0.26901796
SOD1	-0.0889612	0.03582971	0.07763104
AOC3	-0.0705918	0.02705952	0.06117805
LOX	-0.0682892	0.11792244	0.18581718
ATOX1	-0.0657198	0.05452029	0.10500204
PARK7	-0.0511217	0.10282424	0.1670894
S100A13	-0.050502	0.43285367	0.60833488
GPC1	-0.0414486	0.04399324	0.09150595
COMMD1	-0.0226403	0.08013685	0.14369366
S100B	-0.0205516	0.08370808	0.145094

CP	-0.0144214	0.23503534	0.33949549
F5	-0.0091696	0.0492151	0.09843021
AFP	-0.006177	0.52962218	0.70616291
MEMO1	-0.0025619	0.78505882	0.87268967
SLC31A2	-0.0016043	0.97162219	0.97162219
CUTC	-0.0014663	0.7565607	0.87268967
ALB	-0.0002586	0.57999371	0.7380756
TYR	-8.69E-05	0.87934616	0.92371236
AOC1	-4.89E-05	0.91769833	0.93569242
DBH	-3.54E-05	0.78877721	0.87268967
HEPHL1	8.11E-05	0.20653738	0.30685553
S100A5	0.00030837	0.68060346	0.82305535
AOC2	0.00116352	0.58194422	0.7380756
ATP7A	0.00175059	0.8164784	0.88451827
ENOX2	0.00493781	0.6292467	0.77906734
COX17	0.00941984	0.72584635	0.85781842
CUTA	0.01286898	0.88818497	0.92371236
COX11	0.0180965	0.47155816	0.64529012
SCO2	0.02267734	0.09655187	0.16195797
MAP2K1	0.03435245	0.06860074	0.12740138
MT4	0	NA	NA

REFERENCES

1. Frank PG, Lisanti MP. ICAM-1: role in inflammation and in the regulation of vascular permeability. Am J Physiol Heart Circ Physiol. 2008;295(3):H926-H927. eng. Epub 2008/08/08. doi:10.1152/ajpheart.00779.2008. Cited in: Pubmed; PMID 18689494.

2. Sokol CL, Luster AD. The chemokine system in innate immunity. Cold Spring Harb Perspect Biol. 2015;7(5):a016303. eng. doi:10.1101/cshperspect.a016303. Cited in: Pubmed; PMID 25635046.

3. Scheller J, Chalaris A, Schmidt-Arras D, Rose-John S. The pro- and anti-inflammatory properties of the cytokine interleukin-6. Biochimica et Biophysica Acta (BBA) - Molecular Cell Research. 2011 2011/05/01/;1813(5):878-888. doi:https://doi.org/10.1016/j.bbamcr.2011.01.034.

4. Deban L, Russo RC, Sironi M, Moalli F, Scanziani M, Zambelli V, Cuccovillo I, Bastone A, Gobbi M, Valentino S, Doni A, Garlanda C, Danese S, Salvatori G, Sassano M, Evangelista V, Rossi B, Zenaro E, Constantin G, Laudanna C, Bottazzi B, Mantovani A. Regulation of leukocyte recruitment by the long pentraxin PTX3. Nature Immunology. 2010 2010/04/01;11(4):328-334. doi:10.1038/ni.1854.

5. Arlt A, Schafer H. Role of the immediate early response 3 (IER3) gene in cellular stress response, inflammation and tumorigenesis. Eur J Cell Biol. 2011 Jun-Jul;90(6-7):545-52. eng. Epub 2010/11/30. doi:10.1016/j.ejcb.2010.10.002. Cited in: Pubmed; PMID 21112119.

6. de la Fuente H, Cibrián D, Sánchez-Madrid F. Immunoregulatory molecules are master regulators of inflammation during the immune response. FEBS Letters. 2012 2012/08/31/;586(18):2897-2905. doi:https://doi.org/10.1016/j.febslet.2012.07.032.

7. Lopez-Dee Z, Pidcock K, Gutierrez LS. Thrombospondin-1: multiple paths to inflammation. Mediators Inflamm. 2011;2011:296069. eng. Epub 2011/07/19. doi:10.1155/2011/296069. Cited in: Pubmed; PMID 21765615.

8. Ishihara Y, Takemoto T, Itoh K, Ishida A, Yamazaki T. Dual Role of Superoxide Dismutase 2 Induced in Activated Microglia: OXIDATIVE STRESS TOLERANCE AND CONVERGENCE OF INFLAMMATORY RESPONSES. Journal of Biological Chemistry. 2015 September 11, 2015;290(37):22805-22817. doi:10.1074/jbc.M115.659151.

9. Liu T, Zhang L, Joo D, Sun S-C. NF-κB signaling in inflammation. Signal Transduct Target Ther. 2017;2:17023. eng. Epub 2017/07/14. doi:10.1038/sigtrans.2017.23. Cited in: Pubmed; PMID 29158945.

10. Lu J, Chatterjee M, Schmid H, Beck S, Gawaz M. CXCL14 as an emerging immune and inflammatory modulator. Journal of Inflammation. 2016 2016/01/05;13(1):1. doi:10.1186/s12950-015-0109-9.

11. De Langhe E, Aznar-Lopez C, De Vooght V, Vanoirbeek JA, Luyten FP, Lories RJ. Secreted frizzled related proteins inhibit fibrosis in vitro but appear redundant in vivo. Fibrogenesis Tissue Repair. 2014;7:14-14. eng. doi:10.1186/1755-1536-7-14. Cited in: Pubmed; PMID 25317206.

12. Xie T, Wang Y, Deng N, Huang G, Taghavifar F, Geng Y, Liu N, Kulur V, Yao C, Chen P, Liu Z, Stripp B, Tang J, Liang J, Noble PW, Jiang D. Single-Cell Deconvolution of Fibroblast Heterogeneity in Mouse Pulmonary Fibrosis. Cell Rep. 2018 Mar 27;22(13):3625-3640. eng. Epub 2018/03/29. doi:10.1016/j.celrep.2018.03.010. Cited in: Pubmed; PMID 29590628.

13. Leng D, Huan C, Xie T, Liang J, Wang J, Dai H, Wang C, Jiang D. Meta-Analysis of Genetic Programs between Idiopathic Pulmonary Fibrosis and Sarcoidosis. PLOS ONE. 2013;8(8):e71059. doi:10.1371/journal.pone.0071059.

14. Enomoto Y, Matsushima S, Shibata K, Aoshima Y, Yagi H, Meguro S, Kawasaki H, Kosugi I, Fujisawa T, Enomoto N, Inui N, Nakamura Y, Suda T, Iwashita T. LTBP2 is secreted from lung myofibroblasts and is a potential biomarker for idiopathic pulmonary fibrosis. Clin Sci (Lond). 2018 Jul 31;132(14):1565-1580. eng. Epub 2018/07/15. doi:10.1042/cs20180435. Cited in: Pubmed; PMID 30006483.

15. Liu G, Cooley MA, Jarnicki AG, Borghuis T, Nair PM, Tjin G, Hsu AC, Haw TJ, Fricker M, Harrison CL, Jones B, Hansbro NG, Wark PA, Horvat JC, Argraves WS, Oliver BG, Knight DA, Burgess JK, Hansbro PM. Fibulin-1c regulates transforming growth factor-beta activation in pulmonary tissue fibrosis. JCI Insight. 2019 Jul 25;5. eng. Epub 2019/07/26. doi:10.1172/jci.insight.124529. Cited in: Pubmed; PMID 31343988.

16. Venkatesan N, Roughley PJ, Ludwig MS. Proteoglycan expression in bleomycin lung fibroblasts: role of transforming growth factor-β1 and interferon-γ. American Journal of Physiology-Lung Cellular and Molecular Physiology. 2002;283(4):L806-L814. doi:10.1152/ajplung.00061.2002. Cited in: Pubmed; PMID 12225958.

17. Germanguz I, Aranda E, Xiong JC, Kissel N, Nichols A, Gadee E, O'Neill JD. Fibrotic human lung extracellular matrix as a disease-specific substrate for 3D in-vitro models of pulmonary fibrosis. bioRxiv. 2019:833913. doi:10.1101/833913.

18. Decaris ML, Gatmaitan M, FlorCruz S, Luo F, Li K, Holmes WE, Hellerstein MK, Turner SM, Emson CL. Proteomic Analysis of Altered Extracellular Matrix Turnover in Bleomycin-induced Pulmonary Fibrosis. Molecular & Cellular Proteomics. 2014;13(7):1741-1752. doi:10.1074/mcp.M113.037267.

19. Martinez FJ, Collard HR, Pardo A, Raghu G, Richeldi L, Selman M, Swigris JJ, Taniguchi H, Wells AU. Idiopathic pulmonary fibrosis. Nature reviews Disease primers. 2017 Oct 20;3:17074. doi:10.1038/nrdp.2017.74. Cited in: Pubmed; PMID 29052582.

20. Richards TJ, Kaminski N, Baribaud F, Flavin S, Brodmerkel C, Horowitz D, Li K, Choi J, Vuga LJ, Lindell KO, Klesen M, Zhang Y, Gibson KF. Peripheral blood proteins predict mortality in idiopathic pulmonary fibrosis. Am J Respir Crit Care Med. 2012 Jan 1;185(1):67-76. eng. Epub 2011/10/22. doi:10.1164/rccm.201101-0058OC. Cited in: Pubmed; PMID 22016448.

21. Vuga LJ, Milosevic J, Pandit K, Ben-Yehudah A, Chu Y, Richards T, Sciurba J, Myerburg M, Zhang Y, Parwani AV, Gibson KF, Kaminski N. Cartilage oligomeric matrix protein in idiopathic pulmonary fibrosis. PLoS One. 2013;8(12):e83120. eng. Epub 2014/01/01. doi:10.1371/journal.pone.0083120. Cited in: Pubmed; PMID 24376648.

22. Pardo A, Cabrera S, Maldonado M, Selman M. Role of matrix metalloproteinases in the pathogenesis of idiopathic pulmonary fibrosis. Respiratory research. 2016;17:23-23. eng. doi:10.1186/s12931-016-0343-6. Cited in: Pubmed; PMID 26944412.

23. Estany S, Vicens-Zygmunt V, Llatjós R, Montes A, Penín R, Escobar I, Xaubet A, Santos S, Manresa F, Dorca J, Molina-Molina M. Lung fibrotic tenascin-C upregulation is associated with other extracellular matrix proteins and induced by TGFβ1. BMC Pulm Med. 2014;14:120-120. eng. doi:10.1186/1471-2466-14-120. Cited in: Pubmed; PMID 25064447.

24. Wong SLI, Sukkar MB. The SPARC protein: an overview of its role in lung cancer and pulmonary fibrosis and its potential role in chronic airways disease. Br J Pharmacol. 2017;174(1):3-14. eng. Epub 2016/11/25. doi:10.1111/bph.13653. Cited in: Pubmed; PMID 27759879.

25. Sangaletti S, Tripodo C, Cappetti B, Casalini P, Chiodoni C, Piconese S, Santangelo A, Parenza M, Arioli I, Miotti S, Colombo MP. SPARC oppositely regulates inflammation and fibrosis in bleomycin-induced lung damage. The American journal of pathology. 2011;179(6):3000-3010. eng. Epub 2011/10/11. doi:10.1016/j.ajpath.2011.08.027. Cited in: Pubmed; PMID 22001347.

26. Chang W, Wei K, Jacobs SS, Upadhyay D, Weill D, Rosen GD. SPARC suppresses apoptosis of idiopathic pulmonary fibrosis fibroblasts through constitutive activation of beta-catenin. J Biol Chem. 2010 Mar 12;285(11):8196-206. eng. Epub 2010/01/12. doi:10.1074/jbc.M109.025684. Cited in: Pubmed; PMID 20061390.

27. Emblom-Callahan MC, Chhina MK, Shlobin OA, Ahmad S, Reese ES, Iyer EPR, Cox DN, Brenner R, Burton NA, Grant GM, Nathan SD. Genomic phenotype of non-cultured pulmonary fibroblasts in idiopathic pulmonary fibrosis. Genomics. 2010 2010/09/01/;96(3):134-145. doi:https://doi.org/10.1016/j.ygeno.2010.04.005.

28. Selman M, Pardo A, Barrera L, Estrada A, Watson SR, Wilson K, Aziz N, Kaminski N, Zlotnik A. Gene expression profiles distinguish idiopathic pulmonary fibrosis from hypersensitivity pneumonitis. American journal of respiratory and critical care medicine.

2006;173(2):188-198. eng. Epub 2005/09/15. doi:10.1164/rccm.200504-644OC. Cited in: Pubmed; PMID 16166619.

29. Reyfman PA, Walter JM, Joshi N, Anekalla KR, McQuattie-Pimentel AC, Chiu S, Fernandez R, Akbarpour M, Chen C-I, Ren Z, Verma R, Abdala-Valencia H, Nam K, Chi M, Han S, Gonzalez-Gonzalez FJ, Soberanes S, Watanabe S, Williams KJN, Flozak AS, Nicholson TT, Morgan VK, Winter DR, Hinchcliff M, Hrusch CL, Guzy RD, Bonham CA, Sperling AI, Bag R, Hamanaka RB, Mutlu GM, Yeldandi AV, Marshall SA, Shilatifard A, Amaral LAN, Perlman H, Sznajder JI, Argento AC, Gillespie CT, Dematte J, Jain M, Singer BD, Ridge KM, Lam AP, Bharat A, Bhorade SM, Gottardi CJ, Budinger GRS, Misharin AV. Single-Cell Transcriptomic Analysis of Human Lung Provides Insights into the Pathobiology of Pulmonary Fibrosis. American journal of respiratory and critical care medicine. 2019;199(12):1517-1536. eng. doi:10.1164/rccm.201712-2410OC. Cited in: Pubmed; PMID 30554520.

SUPPLEMENTAL FIGURE LEGENTS

Figure S1. (A) Histology of the tissue used for sequencing showing preserved lung architecture in the control lungs, extensive scar tissue in the IPF lower lobes and a relatively unaffected histology in two of the IPF upper lobe samples. Age and gender are listed following the sample ID name. **(B)** tSNE plot of lung cells colored by sample ID.

Figure S2. tSNE plot of lung cells showing fibroblast Cluster-5, neighboring B-cell cluster. This cluster was found to contain cells expressing mainly HLA type II family genes and was excluded from our fibroblast analysis.

Figure S3. tSNE plot of lung fibroblasts colored by sample ID.

Figure S4. UMAP plot depicting alveolar and adventitial fibroblasts **(A)** and CTHRC1+ fibroblasts **(B).**

Figure S5. UMAP_1 plots of lung fibroblasts after re-analysis using raw data from a published IPF study (Reyfman et al., 2019). (**A**) UMAP_1 plot showing fibroblast sub-cluster 2 (green) with distinct sub-populations originating from Control, and IPF samples as well as a mixed Control/IPF subpopulation. (**B**) UMAP_1 plot showing the contribution of each sample type in the different fibroblast sub-clusters.

Figure S6. **MOXD1 detection in cultured human lung fibroblasts.** Cells were isolated from different donors (donor replicas). Adjacent IPF_UP and IP_LOW images show cells isolated from the same donor's upper and lower lung lobes respectively. This is a high-resolution image of Figure 8D.

Figure S7. **MOXD1 detection in human lung cells.** Cells were isolated as in Fig. S6. This image includes IHC staining. (A) MOXD1 immunofluorescence staining in lung parenchyma frozen tissue, obtained from age-matched control and IPF lungs (x20 magnification, 50μm). Red: MOXD1; green: α-SMA (myofibroblast marker). (B) IHC staining of MOXD1 in lung parenchyma tissue from patients with age-matched control and IPF. Yellow arrows highlight the MOXD1 expression in fibroblast foci (x20 magnification, 50μm).
