# Supplementary material for: Early events marking lung fibroblast transition to profibrotic state in idiopathic pulmonary fibrosis
Source: Respir Res. 2023 Apr 21;24:116. doi: 10.1186/s12931-023-02419-0 (PMC10122312; doi:10.1186/s12931-023-02419-0)
Supplement: Supplementary file 2 — Additional file 2. Supplementary Figures. [file 12931_2023_2419_MOESM2_ESM.pdf]

Jia *et al.*, "*Early events marking lung fibroblast transition to profibrotic state in idiopathic pulmonary fibrosis*"

SUPPLEMENTAL FIGURES

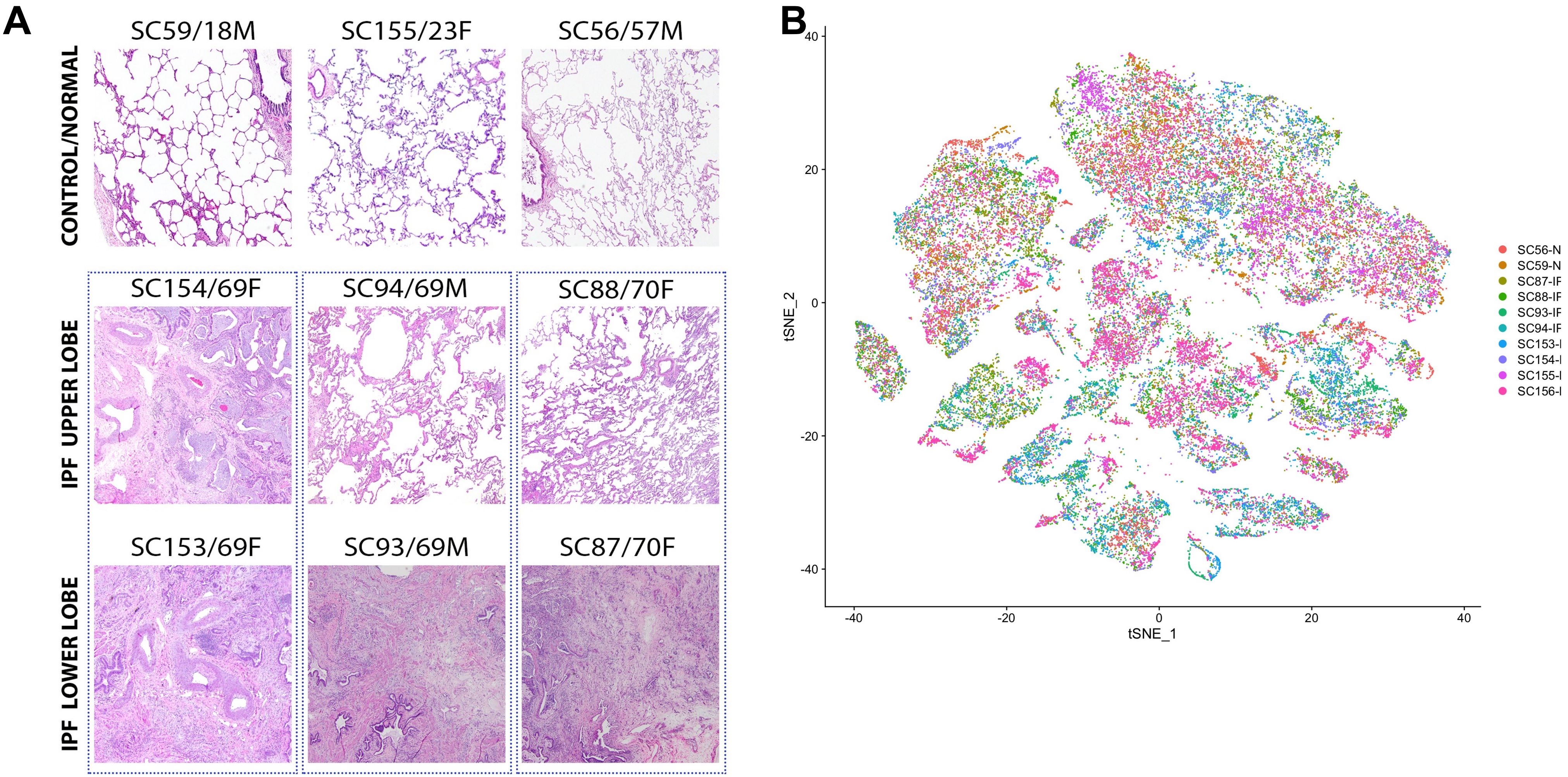

A
SC59/18M
SC155/23F
SC56/57M
CONTROL/NORMAL
SC154/69F
SC94/69M
SC88/70F
IPF UPPER LOBE
SC153/69F
SC93/69M
SC87/70F
IPF LOWER LOBE
B
tSNE_1
tSNE_2
SC56-N
SC59-N
SC87-IF
SC88-IF
SC93-IF
SC94-IF
SC153-I
SC154-I
SC155-I
SC156-I

FIGURE S1

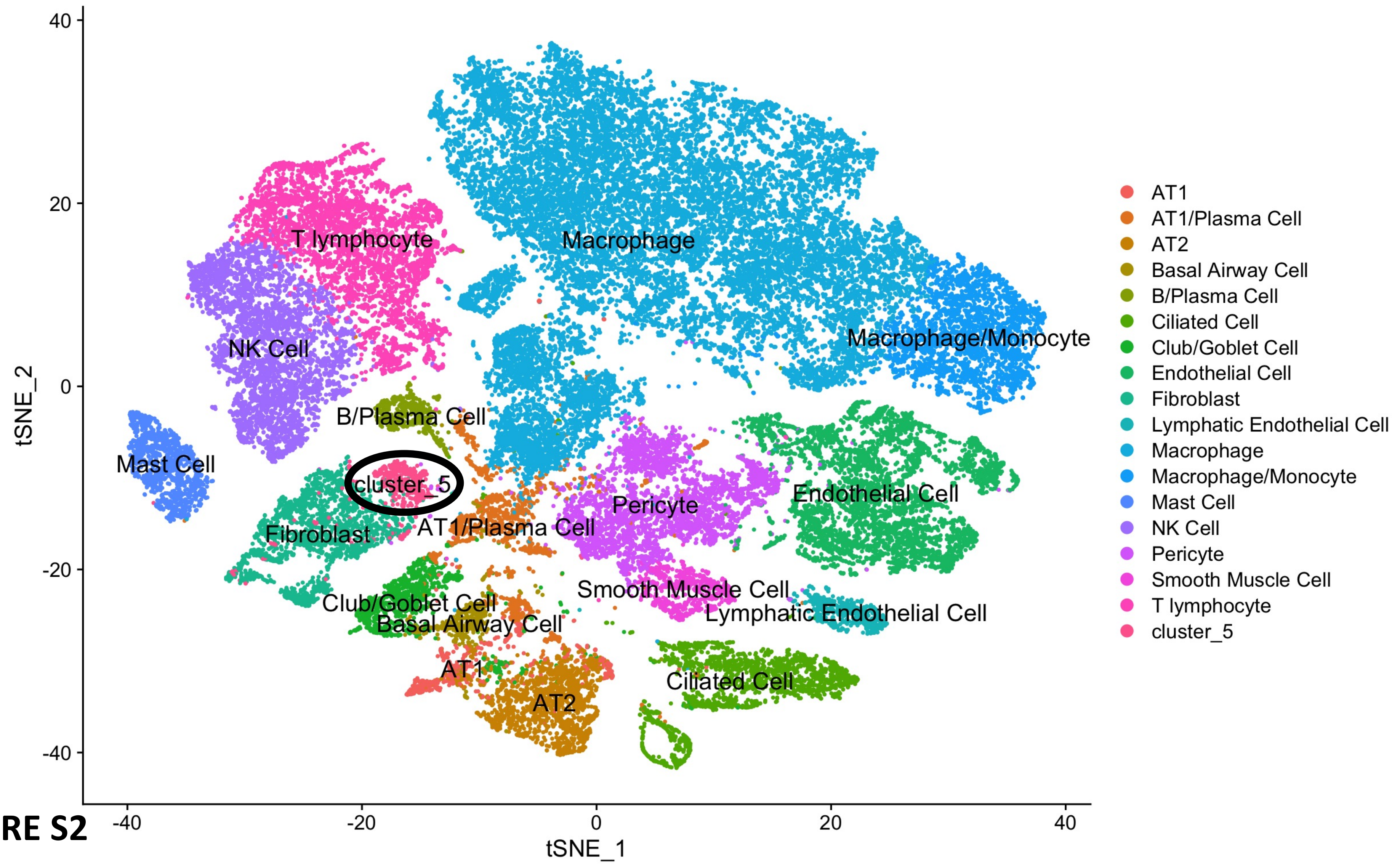
T lymphocyte
Macrophage
NK Cell
Macrophage/Monocyte
B/Plasma Cell
Mast Cell
cluster_5
Pericyte
Endothelial Cell
Fibroblast
AT1/Plasma Cell
Smooth Muscle Cell
Club/Goblet Cell
Lymphatic Endothelial Cell
Basal Airway Cell
AT1
Ciliated Cell
AT2
tSNE_2
tSNE_1
40
20
0
-20
-40
AT1
AT1/Plasma Cell
AT2
Basal Airway Cell
B/Plasma Cell
Ciliated Cell
Club/Goblet Cell
Endothelial Cell
Fibroblast
Lymphatic Endothelial Cell
Macrophage
Macrophage/Monocyte
Mast Cell
NK Cell
Pericyte
Smooth Muscle Cell
T lymphocyte
cluster_5

FIGURE S2

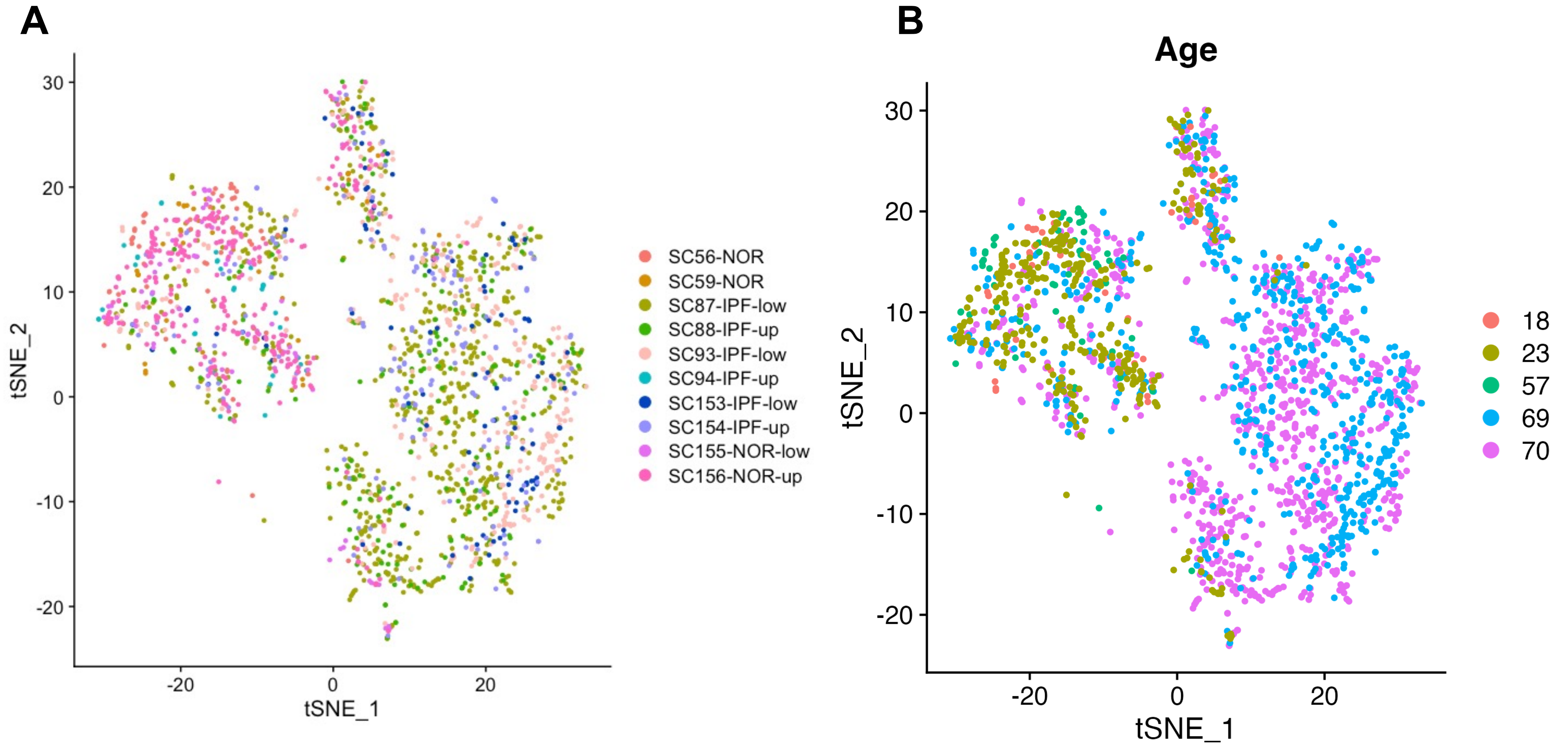
A
tSNE_2
tSNE_1
30
20
10
0
-10
-20
-20
0
20
SC56-NOR
SC59-NOR
SC87-IPF-low
SC88-IPF-up
SC93-IPF-low
SC94-IPF-up
SC153-IPF-low
SC154-IPF-up
SC155-NOR-low
SC156-NOR-up
B
Age
tSNE_2
tSNE_1
30
20
10
0
-10
-20
-20
0
20
18
23
57
69
70

FIGURE S3

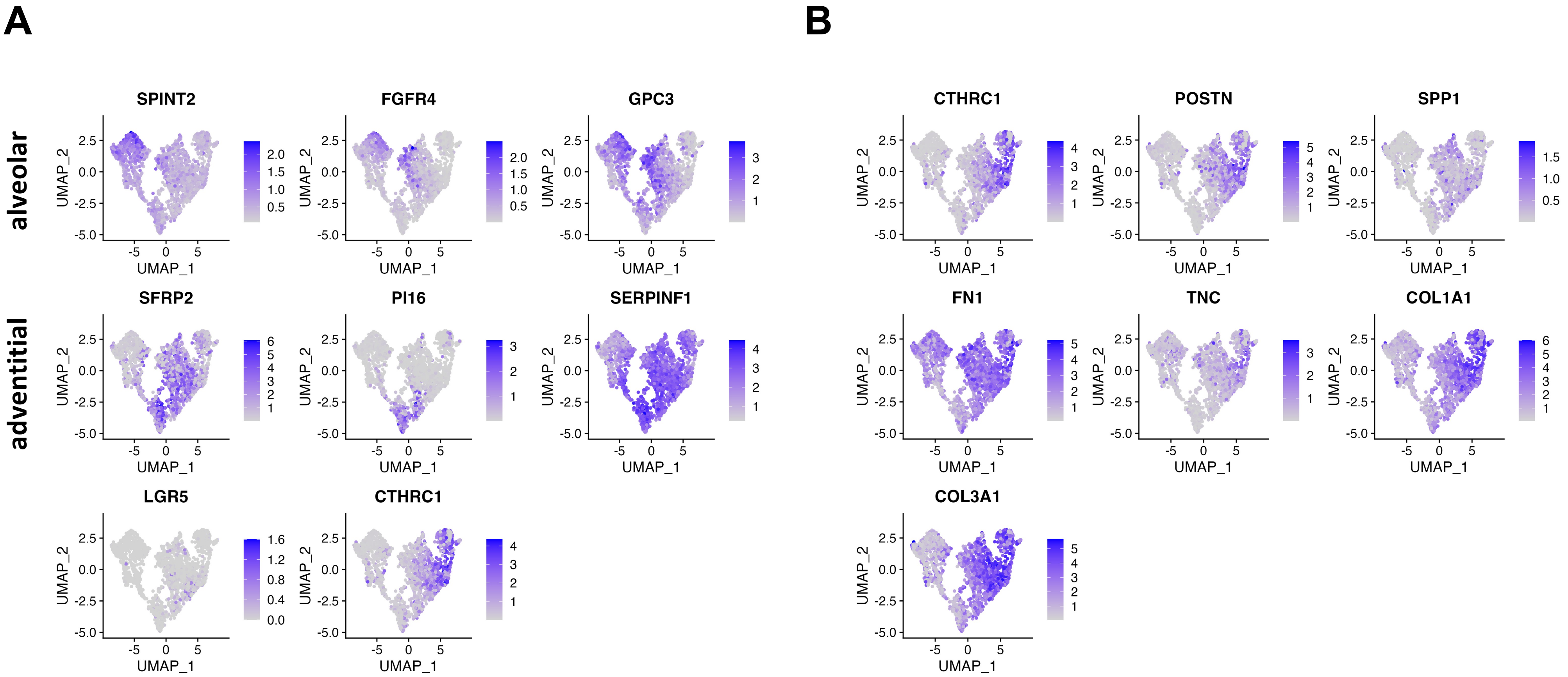
A
B
alveolar
adventitial
SPINT2
FGFR4
GPC3
SFRP2
PI16
SERPINF1
LGR5
CTHRC1
CTHRC1
POSTN
SPP1
FN1
TNC
COL1A1
COL3A1
UMAP_1
UMAP_2

FIGURE S4

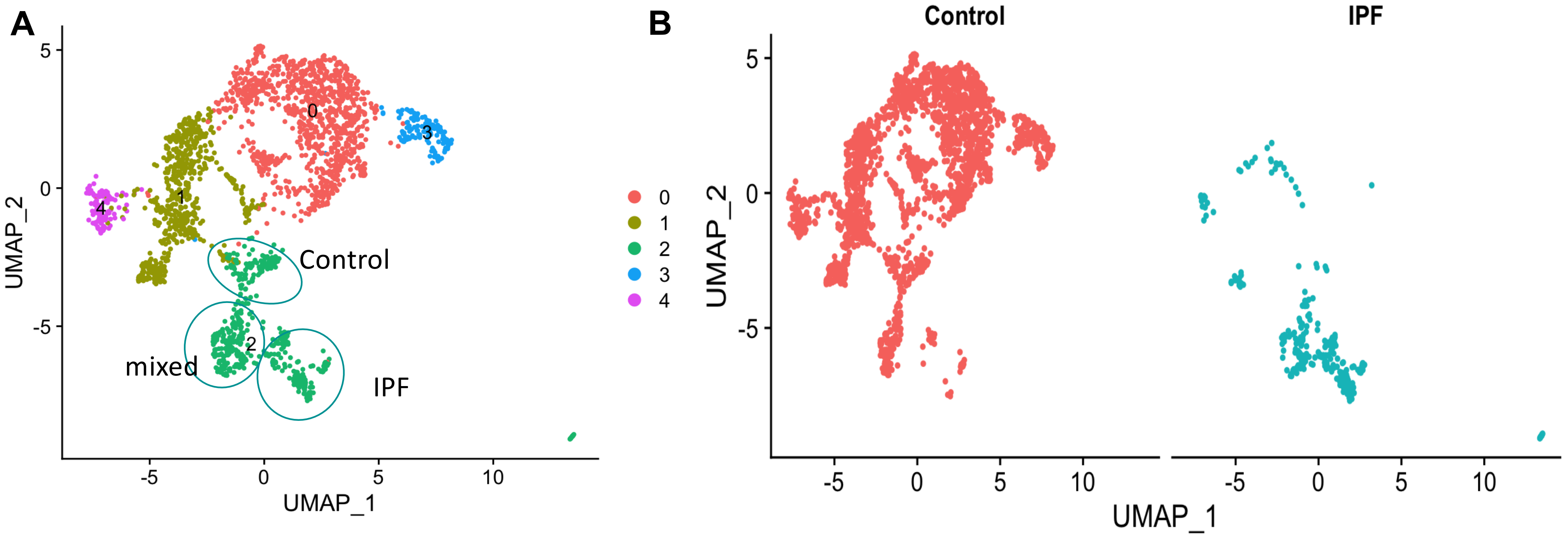

FIGURE S5

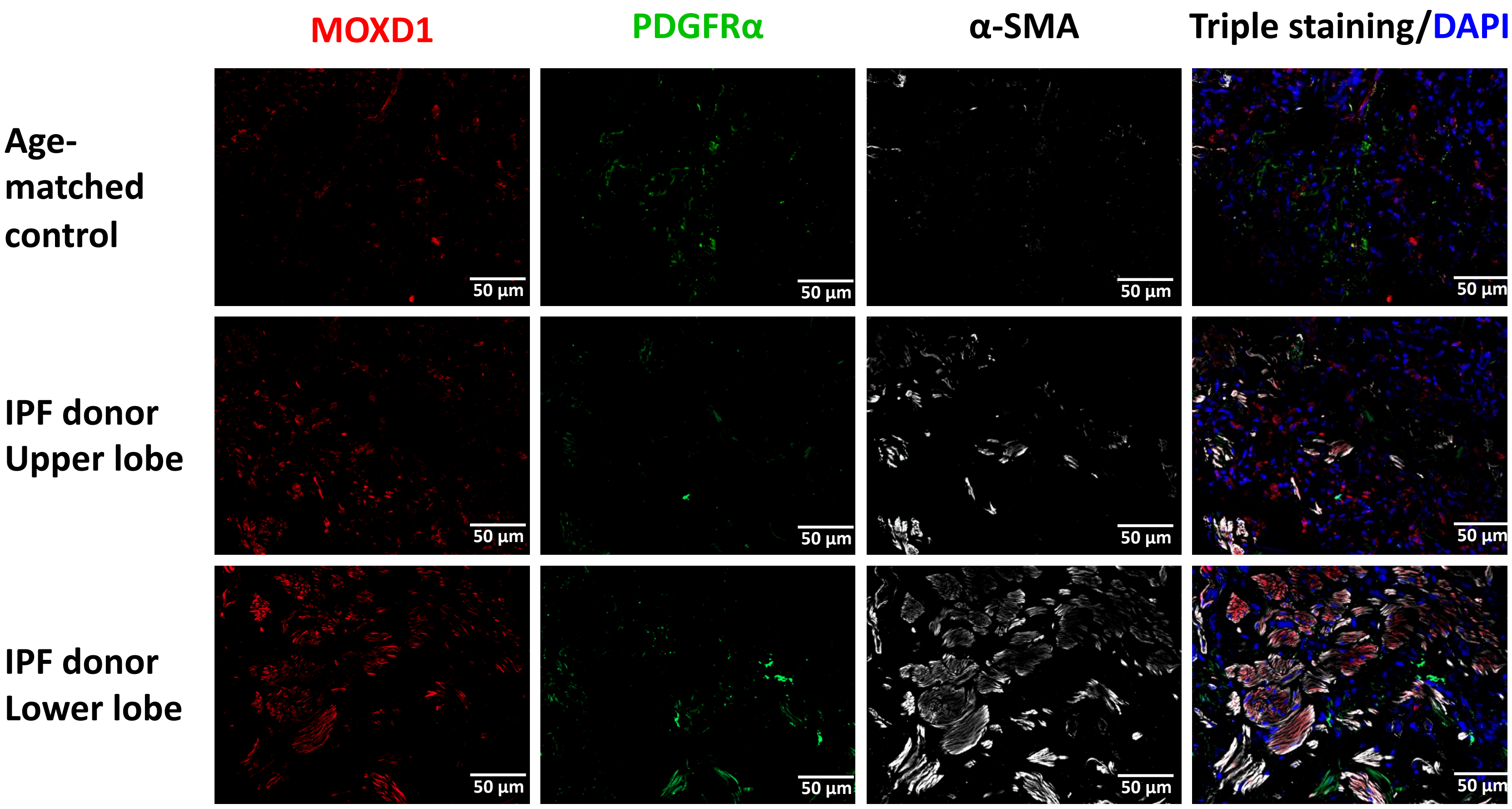
MOXD1
PDGFRα
α-SMA
Triple staining/DAPI
Age-matched control
IPF donor Upper lobe
IPF donor Lower lobe
50 µm

FIGURE S6

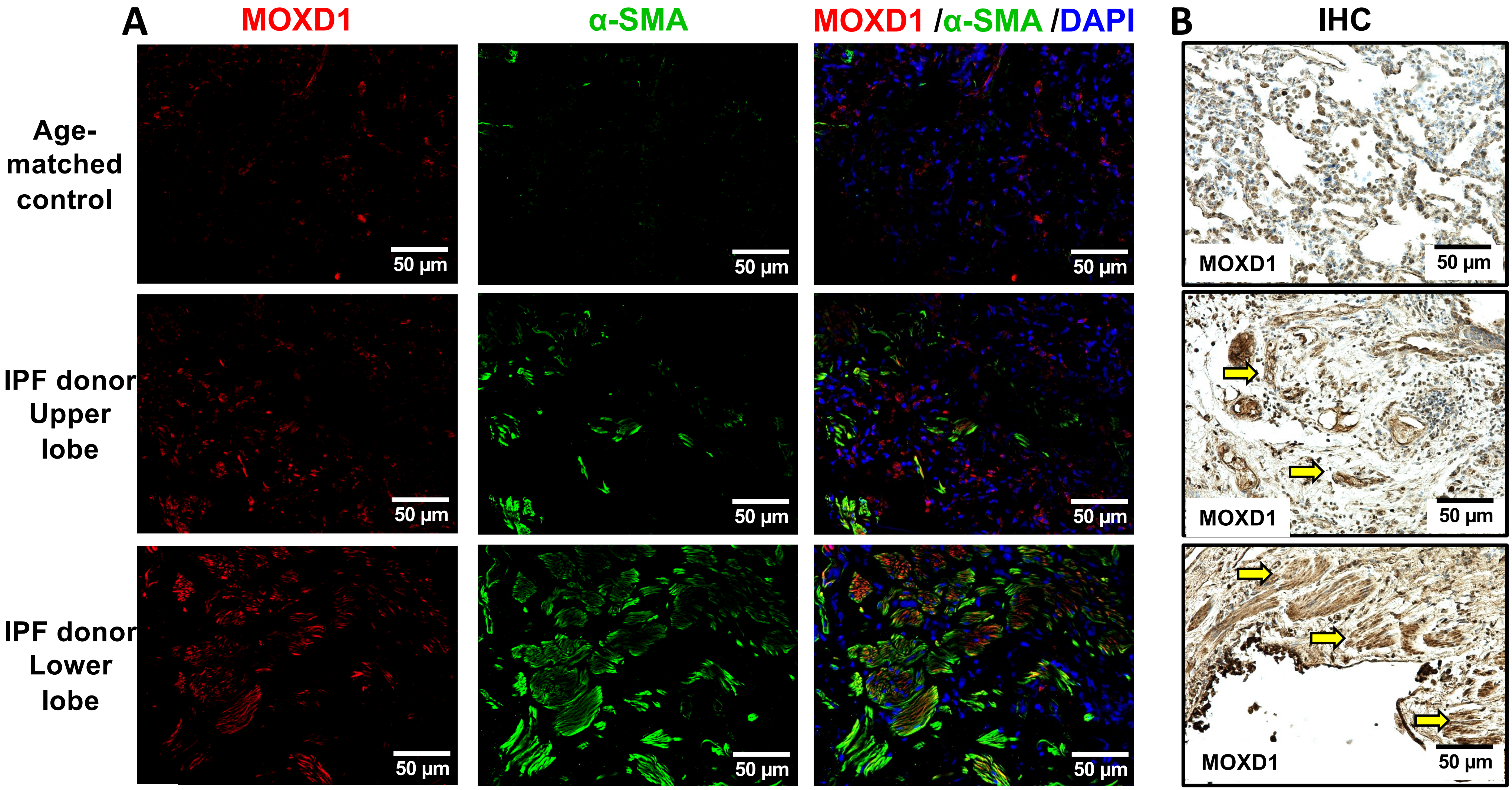
A
MOXD1
α-SMA
MOXD1 /α-SMA /DAPI
B
IHC
Age-matched control
IPF donor Upper lobe
IPF donor Lower lobe
50 μm
MOXD1

FIGURE S7
